# LRRK2 Mutation Alters Dopamine D2 Receptor Localization in Induced Pluripotent Stem Cells‐Derived Astrocytes From Parkinson's Disease Patients: Implications for Neuronal Damage

**DOI:** 10.1111/jnc.70510

**Published:** 2026-07-01

**Authors:** Veronica Mutti, Federica Bono, Zaira Tomasoni, Dounia Fadel, Chiara Gnutti, Giulia Sbrini, Carolina Gaudenzi, Valentina Salvi, Daniela Bosisio, Isabella Russo, Silvia Bolognin, Jens C. Schwamborn, Cristina Missale, Chiara Fiorentini

**Affiliations:** ^1^ Department of Molecular and Translational Medicine University of Brescia Brescia Italy; ^2^ MERLN Institute for Technology‐Inspired Regenerative Medicine Maastricht University Maastricht the Netherlands; ^3^ Developmental and Cellular Biology, Luxembourg Centre for Systems Biomedicine University of Luxembourg Belvaux Luxembourg

**Keywords:** astrocytes, dopamine D2 receptors, dopaminergic neurons, induced pluripotent stem cells, LRRK2, neuroinflammation, Parkinson's disease

## Abstract

Astrocytes, the most abundant glia subtype, exert a wide range of functions, many of which are essential for maintaining neuronal homeostasis. A variety of neurotransmitter receptors are expressed on astrocytes allowing them to sense extracellular signals and respond by releasing neuroactive mediators. Among them, a wide variety of G protein‐coupled receptors have been detected, including those for dopamine (DA), known to play a major role in modulating astrocytic activity. Evidence that astrocytic DA D2 receptors (D2R) increase the release of trophic factors and suppress neuroinflammation has been provided. Thus, DA signaling in astrocytes may be crucially involved in the mechanisms underlying the degeneration of DA neurons in Parkinson's disease (PD). In this study, human astrocytes were generated from induced pluripotent stem cell (iPSC) lines derived from two PD patients bearing G2019S LRRK2 kinase activating mutation. The effect of the PD‐related mutation in astrocytes was analyzed, focusing on DA receptor's expression and localization. As expected, astrocytes carrying G2019S mutation in LRRK2 displayed a reactive phenotype with increased secretion of inflammatory cytokines and reduced ability to support DA neurons' trophism in astrocytes/neurons co‐culture experiments. Intriguingly, PD astrocytes exhibited reduced membrane expression of D2R. Inhibiting the abnormally increased kinase activity was able to revert the PD astrocytes' reactive phenotype and to rescue the D2R membrane localization. We thus provide new insights into how G2019S mutation in LRRK2, by disrupting the astrocytic physiological localization of D2R, may impair protective DA signaling, resulting in increased neuroinflammation and neuronal damage.

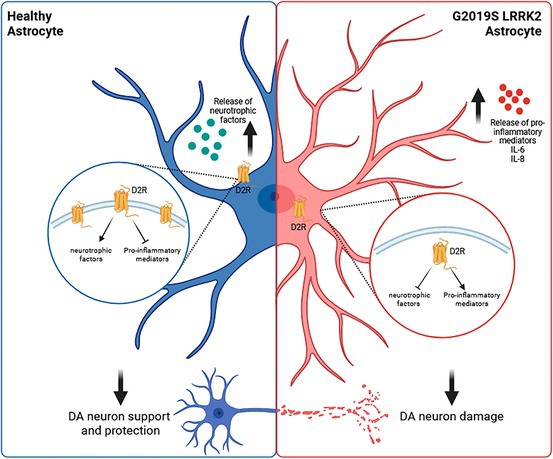

## Graphical abstract

In this study, human astrocytes were obtained from induced pluripotent stem cells (iPSCs) of Parkinson's disease (PD) patients carrying the G2019S LRRK2 mutation, the most common genetic cause of PD. These astrocytes display a reactive and inflammatory profile, with increased GFAP levels, elevated cytokine release, and reduced support for dopaminergic neurons. Importantly, the trafficking of dopamine D2 receptors (D2R) to the cell membrane is impaired, compromising their protective signaling functions, including the regulation of neurotrophic factor release and inflammation. Notably, inhibiting the hyperactive LRRK2 kinase restores D2R membrane localization and function, rescuing both trophic support and anti‐inflammatory responses. These findings highlight a mechanism by which LRRK2 mutations in astrocytes may contribute to neuronal vulnerability in PD. Created with Biorender.com.

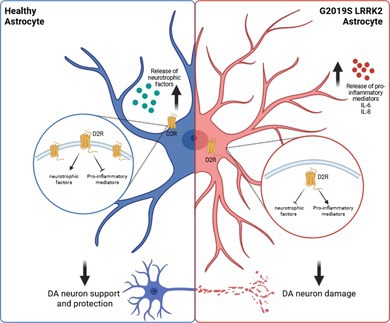

**Abbreviations**
cDNA: complementary deoxyribonucleic acid
D1R: dopamine D1 receptor
D2R: dopamine D2 receptor
DA: dopamine
DMEM: dulbecco's modified eagle medium
GPCR: G protein‐coupled receptors
IL: interleukin
iPSC: induced pluripotent stem cell
LRRK2: Leucine‐Rich Repeat Kinase 2
LSM: laser scanning microscope
PBS: phosphate buffered saline
PCR: polymerase chain reaction
PD: Parkinson's disease
RNA: ribonucleic acid
RRID: Research Resource Identifier
RT: room temperature

## 1 Introduction

Astrocytes are the most abundant glial cells, uniformly distributed throughout the central nervous system, with a variety of functions that include neuronal support mainly achieved by supplying neurotrophic factors and antioxidants (Miyazaki and Asanuma 2020). Moreover, astrocytes are involved in the control of inflammatory responses since reactive astrocytes release numerous inflammatory mediators with both detrimental and neuroprotective potentials (Hauser and Cookson 2011; Ho et al. 2025; Miyazaki and Asanuma 2020). Therefore, astrocytes have emerged as key elements in most neurodegenerative diseases, where a combination of different pathogenetic mechanisms, including neuroinflammation, oxidative stress, and neuron vulnerability is thought to contribute to neuronal damage. Among them, Parkinson's disease (PD) is a progressive neurodegenerative disorder primarily characterized by the loss of dopaminergic (DA) neurons in the pars compacta of the substantia nigra, which leads to motor impairments, such as bradykinesia, rigidity, and tremor that represent the predominant clinical features of the disease (Kalia and Lang 2015; Tysnes and Storstein 2017). Both environmental and genetic factors are crucial for PD etiology. A subset of patients, in fact, exhibits gene mutations, such as those in the Leucine‐Rich Repeat Kinase 2 (LRRK2) gene, involved in the pathogenesis of both sporadic and familial PD. LRRK2 encodes a ubiquitously expressed protein with both GTPase and kinase domains, with many functions including regulation of the autophagy/lysosomal pathway, cytoskeletal organization, intracellular vesicle trafficking, and maintenance of mitochondrial integrity and function (Bailey and Cookson 2024). The most common pathogenic mutation in the LRRK2 gene is G2019S that occurs in the kinase domain and increases the enzyme catalytic activity, thus inducing DA neuron dysfunctions that contribute to degeneration (Thakur et al. 2022; Weykopf et al. 2019). Interestingly, non‐neuronal cells, such as astrocytes, express high levels of LRRK2 (Ho et al. 2025) and evidence showing that astrocytes with abnormal LRRK2 activity may also contribute to DA neuron degeneration has been provided in PD animal models and post‐mortem brain tissue (Booth et al. 2019; Bailey and Cookson 2024). Moreover, additional evidence has been obtained by using the induced pluripotent stem cell (iPSC) technology and iPSC lines derived from PD patients to obtain in vitro models of human astrocytes carrying G2019S mutation in LRRK2 (de Rus Jacquet et al. 2021; Di Domenico et al. 2019; Ho et al. 2024; Pons‐Espinal et al. 2024; Ramos‐Gonzalez et al. 2021). The analysis of iPSC‐derived astrocytes has shown that G2019S mutation induced a significant impairment in the endolysosomal system (Erb and Moore 2020), including defects in the chaperone‐mediated autophagy, macroautophagy, and protein degradation pathways, thus leading to intracellular pathological accumulation of extracellular alpha‐synuclein (α‐syn) (Di Domenico et al. 2019). Moreover, human astrocytes carrying G2019S mutation in LRRK2 were characterized by an increased production of proinflammatory cytokines, such as interleukin IL‐1β, IL‐6, and TNF‐α, and an inability to release neurotrophic factors (de Rus Jacquet et al. 2021; Pons‐Espinal et al. 2024), together with an altered morphology and mitochondrial dynamics (Pons‐Espinal et al. 2024).

Recently, we used iPSC lines derived from two PD patients carrying the G2019S LRRK2 mutation together with their isogenic counterparts, in which the mutation has been corrected (Reinhardt et al. 2013), to generate DA neurons (Bono et al. 2021). As reported, the analysis of iPSC‐derived G2019S LRRK2 DA neurons has shown that they exhibited morphological impairments and increased levels of α‐syn aggregates along with abnormalities in receptor trafficking and membrane localization of the DA D3 and the nicotinic acetylcholine receptors, crucial for DA neuron health (Bono et al. 2021). In this study, we generated an almost pure population of human astrocytes from a control iPSC line, followed by their cellular, molecular, and functional characterization. Cultures of human astrocytes were also generated from iPSC lines derived from the two PD patients carrying the G2019S LRRK2 mutation and fully characterized. In particular, in iPSC‐derived astrocytic cultures, the expression and localization of DA receptors were analyzed since several lines of evidence have shown that in astrocytes DA signaling, through the DA D2 receptor (D2R), modulates both the synthesis and release of neurotrophic factors, such as brain‐derived neurotrophic factor (BDNF), nerve growth factor (NGF), and glial cell line‐derived neurotrophic factor (GDNF), and the inflammatory responses (Favetta and Bubacco 2025). Thus, D2R in astrocytes may represent a crucial molecular entity for neuronal protection and survival.

## 2 Materials and Methods

### 2.1 Chemicals and Materials

Accutase (cat. n° A6964), L‐glutamine (cat. n° G7513), poly(D)‐lysine (cat. n° P1024), laminin (cat. n° L2020), paraformaldehyde (PFA) (cat. n° P6148), Triton X‐100 (cat. n° X100), bovine serum albumin (BSA; cat. n° A7030), 3‐3′‐diaminobenzidine (DAB; cat. n° D8001), 4′,6‐diamidino‐2‐phenylindole (DAPI; cat. n° D9542), biotin 3‐sulfo‐N‐hydroxysuccinimide ester sodium salt (sulfo‐NHS‐biotin; cat n° B5161), Tween‐20 (cat n° P7949) and streptavidin agarose resin (cat. n° 20 353) were purchased from Merk Life Science. StemFlex medium (cat. n° 15 627 578), Advance DMEM/F12 medium (cat. n° 12 634 010), Neurobasal medium (cat. n° 21 103 049), N2 (cat. n° 17 502 048) and B27 supplement (cat. n° A3582801), and Glutamax (cat. n° 35 050 061) were from Gibco, Invitrogen (Carlsbad, CA, USA). LDN193189 (cat. n° 04‐0074) was purchased from Stemgent (Cambridge, MA). The Y27632 dihydrochloride (ROCK inhibitor; cat. n° 1254), SB 431542 (cat. no 1614), SCH23990 (cat. n° 0925), sulpiride (cat. n° 0895) and the LRRK2 inhibitor GSK2578215A (cat. n° 4629) were purchased from Tocris Bioscience. The fibroblast growth factor 2 (FGF2; cat. n° 100‐18C), the epidermal growth factor (EGF; cat n° AF10015), the leukemia inhibitory factor (LIF; cat. n° 300‐05) and the ciliary neurotrophic factor (CNTF; cat. n° 450‐13) were purchased from PeproTech. MLi‐2 (cat. n° HY‐100411) was purchased from Med Chem Express.

### 2.2 Human iPSC‐Derived Astrocytes Differentiation

iPSC derived from two patients carrying G2019S LRRK2 mutation (LRRK2‐PD‐1 and LRRK2‐PD‐2; *n* = 2 independent PD patients' lines) as well as from gene‐corrected iPSC of the same patients (LRRK2‐ISO‐1 and LRRK2‐ISO‐2; *n* = 2 independent PD patients' lines), and healthy control iPSC line (CTRL; *n* = 1), all generated and fully characterized by Reinhardt et al. (2013) (Table S1) were used. Informed consent was obtained from all patients and from healthy donors involved in our study, prior to cell donation (Reinhardt et al. 2013).

Cells were differentiated using a previously published protocol (Perriot et al. 2021) with minor modifications. Briefly, iPSC lines were cultured in feeder‐free conditions in Stemflex medium on Matrigel (BD; cat. n° CLS354234) coated culture dishes. For neural induction, iPSC lines were cultured for 24 h in Stemflex medium, in the presence of the ROCK inhibitor, Y27632 (10 μM). After 24 h, culture medium was switched to Neural Induction Medium (NIM) consisting of Advanced DMEM/F12, supplemented with 1% N2, 1% B27, 1% Glutamax, in the presence of 100 nM LDN193189, 20 μM SB 431542 and 4 ng/mL FGF2. After 13 days, neural stem cells (NSC) were dissociated with Accutase and cultured on Matrigel‐coated culture dishes with Neural Expansion Medium (NEM) consisting of Advanced DMEM/F12, supplemented with 1% N2, 1% B27, 1% Glutamax in the presence of 10 ng/mL FGF2 and 10 ng/mL EGF for 2 weeks. Exposure of cells for an additional 2 weeks to Advanced DMEM/F12 supplemented with 10 ng/mL LIF and 10 ng/mL EGF accelerated the switch toward glial cell differentiation, resulting in a proliferative population of astrocyte precursor cells (APCs). For astrocytes terminal differentiation, APCs were dissociated using Accutase and cultured for 4 weeks in Advanced DMEM/F12 supplemented with 1% B27 and 1% Glutamax in the presence of 20 ng/mL CNTF. During the differentiation process, cells were passaged every 3–5 days using Accutase.

At the end of differentiation (Day 70), mature astrocytes were cultured in Advanced DMEM/F12 supplemented with 1% B27 and 1% Glutamax without CNTF.

Conditioned medium (CM) was obtained from iPSC‐derived astrocyte cultures (Day 70) by adding fresh medium and collecting it after 5 days.

In another set of experiments, astrocytes derived from LRRK2‐PD iPSC lines (Day 70) were cultured for 10 days in the absence or in the presence of the GSK2578215A LRRK2 inhibitor (GSK; 200 nM) or the MLi‐2 LRRK2 inhibitor (MLi‐2; 500 nM) (Fell et al. 2015) and analyzed for morphological, biochemical, and molecular parameters, as described below.

### 2.3 Primary Cultures of Mouse Midbrain Neurons

CD1 wild‐type mice were obtained from Charles River Laboratory (Calco, Italy; RRID: IMSR_CRL:22). Animals were bred and housed in the animal facility of the University of Brescia under standard conditions, with ad libitum access to food and water and a 12‐h light/dark cycle. Animal care and use were in accordance with Directive 2010/63/EU. To obtain primary cultures of midbrain neurons, time‐mated pregnancies were established with an accuracy of ±0.5 days. The presence of a vaginal plug, corresponding to embryonic Day 0.5 (E0.5), was checked the morning after mating and used to determine pregnancy status and embryonic developmental stage. All efforts were made to minimize animal suffering and to reduce the number of animals used. Colony maintenance and embryo collection procedures were approved by the Institutional Animal Care and Use Committee (IACUC) of the University of Brescia and complied with Italian regulations (Italian Ministry of Health authorization no. 211B5.N.E9V). Primary cultures of midbrain neurons were prepared from wild‐type mice (*n* = 15), sacrificed by cervical dislocation, by dissecting the ventral mesencephalon from E12.5 mouse embryos, as previously described (Bontempi et al. 2017; Sbrini et al. 2024). Briefly, embryos were decapitated using sharp surgical scissors, and mesencephalons were collected, mechanically dissociated at room temperature, and resuspended in NB27 medium, composed of Neurobasal medium supplemented with 2 mM L‐glutamine and 2% B27 supplement. Cells were counted and seeded on poly(D)‐lysine/laminin‐coated plates (1 × 10^5^ cells/well) at 37°C in a humidified 5% CO_2_ atmosphere. The medium was partially changed every 2 days, and all pharmacological treatments were performed 7 days after seeding.

### 2.4 Midbrain Neuron/Astrocytes Co‐Culture Experiments and Treatments

iPSC‐derived astrocytes from CTRL, LRRK2‐PD‐1, and LRRK2‐PD‐2 lines, as well as the corresponding gene‐corrected LRRK2‐ISO‐1 and LRRK2‐ISO‐2 lines (Day 70), were plated in 12‐well plates pre‐coated with Matrigel at a density of 3 × 10^4^ cells per well. Cells were cultured for 7 days in Advanced DMEM/F12 supplemented with 1% B27 and 1% GlutaMAX until confluence was reached.

Primary midbrain neuron cultures were prepared from wild‐type mice, as described above, and seeded at a density of 1.5 × 10^5^ cells per well either on confluent monolayers of iPSC‐derived astrocytes or on poly‐D‐lysine/laminin‐coated wells. In parallel experiments, primary midbrain neurons were seeded on poly‐D‐lysine/laminin‐coated wells and exposed to conditioned medium collected from iPSC‐derived astrocyte cultures, as described above.

All cultures were maintained in NB27 medium at 37°C in a humidified atmosphere containing 5% CO_2_ for 7 days. Cells were then fixed and morphologically analyzed by immunocytochemistry, as described below.

In a separate set of experiments, midbrain neurons were seeded on confluent monolayers of CTRL iPSC‐derived astrocytes (Day 70) or on poly‐D‐lysine/laminin‐coated wells and cultured for 5 days in the absence or presence of the D1R antagonist SCH23990 (10 μM) or the D2R antagonist sulpiride (5 μM) in NB27 medium at 37°C in a humidified atmosphere containing 5% CO_2_. Cultures were subsequently fixed and morphologically analyzed by immunocytochemistry, as described below.

In another set of experiments, midbrain neurons were seeded on confluent monolayers of iPSC‐derived astrocytes from LRRK2‐PD lines, previously untreated or treated with LRRK2 inhibitors, GSK (200 nM) or MLi‐2 (500 nM) for 10 days as described above. Cultures were maintained in NB27 medium, in the presence or in the absence of LRRK2 inhibitors, and cultured for 7 days at 37°C in a humidified atmosphere containing 5% CO_2_, before fixation and immunocytochemical analysis, as described below. To exclude direct neuronal effects, parallel experiments were performed by incubating mouse midbrain neurons for 7 days with the same concentration of both GSK and MLi‐2.

### 2.5 Immunocytochemistry and Morphological Analyses

Midbrain neuron cultures were fixed using 4% PFA for 10 min and incubated in 3% hydrogen peroxidase (Sigma Aldrich, cat. n° 88 597) for 10 min to inhibit endogenous peroxidase activity. Cells were then blocked in PBS (Sigma Aldrich, cat. n° P4417) containing 0.1% Triton X‐100 and 5% BSA and incubated with the rabbit anti‐TH polyclonal antibody, to detect dopaminergic (DA) neurons (1:700) (Table S2) at 4°C overnight. The next day, cells were incubated at room temperature (RT) with a biotinylated secondary antibody (Vector, cat. n° BA‐1000) followed by DAB chromogen incubation. Digital images of the immunocytochemical assays were captured with an Olympus IX51 microscope connected to an Olympus digital camera. Morphometric measurements were performed on digitalized images using NIH ImageJ software (NIH, Bethesda, MD, USA). The morphologic indicators of structural plasticity were the maximal dendrite length and the number of primary dendrites, as previously described (Bono et al. 2018; Mutti et al. 2022; Sbrini et al. 2024). Three slides per treatment group were examined to obtain measurements from at least 30 TH‐positive neurons. Cells were randomly selected, and all analyses were performed blind to both experimental conditions and treatments.

### 2.6 Fluorescence Microscopy and Image Analysis

Astrocytes derived from CTRL, LRRK2‐PD‐1 and LRRK2‐PD‐2 and the relative gene‐corrected LRRK2‐ISO‐1 and LRRK2‐ISO‐2 iPSC lines, at different time‐points of the differentiation process, were fixed with 4% PFA for 10 min, blocked in PBS containing 0.1% Triton X‐100 and 5% BSA, and incubated overnight at 4°C with the following primary antibodies: anti‐SOX2 (1:100), anti‐Nestin (1:400), anti‐MAP2 (1:400), anti‐GFAP (1:900), anti‐S100β (1:100), anti‐GLAST (1:500), anti‐pan‐Cadherin (1:200), anti‐D1R (1:100), and anti‐D2R (1:50) (Table S2). Cells were then incubated for 30 min at room temperature with appropriate Alexa Fluor 488‐ and 594‐conjugated secondary antibodies (Jackson Immuno Research) (Table S3). Nuclei were stained with DAPI. Confocal z‐stack images were acquired using a Zeiss LSM 880 confocal microscope equipped with a Plan‐Apochromat 63×/1.4 numerical aperture oil objective, and processed with Zen 2.3 Software (Carl Zeiss AG, Oberkochen, Germany). The proportion of GFAP‐positive cells was determined by counting the number of GFAP‐positive cells relative to the total number of nuclei identified by DAPI staining.

GFAP fluorescence was quantified by calculating the corrected total cell fluorescence (CTCF) for each astrocyte, using the NIH ImageJ software (NIH, Bethesda, MD, USA). Briefly, for each cell, a region of interest (ROI) was manually drawn to outline the cell body. Then, the integrated density (sum of pixel values within the ROI) and ROI area were measured. Three background ROIs were selected from cell‐free areas of the same image to determine the mean background fluorescence. The CTCF was calculated according to the following formula: *CTCF = integrated density − (cell area × mean background fluorescence)*.

To quantify the territory size of individual astrocytes, defined as the area occupied by their processes, including the fine terminal extensions of the main branches, the distal tips of the astrocytic processes were connected with straight lines to form a polygon outlining the cell's territory. The area of this polygon was then measured using ImageJ software.

Colocalization analysis was performed using the Coloc2 plugin in Fiji, based on ImageJ. Confocal images acquired in two channels were analyzed by selecting representative regions of interest (ROIs) corresponding to the plasma membrane of individual astrocytes. Colocalization between the D1R‐ or D2R‐signal (channel 1) and the plasma membrane marker pan‐cadherin (channel 2) was quantified using Manders' coefficients. In particular, thresholded Manders' coefficients (M1 and M2) were used, calculated after applying Costes' automatic thresholding approach to reduce background noise and non‐specific signal contribution. The M1 coefficient represents the fraction of D1R or D2R signal overlapping with pan‐cadherin‐positive regions, and values range from 0 to 1, where 1 indicates complete signal overlap. For all image analyses, at least 30 randomly selected cells per coverslip were evaluated for each experimental condition, derived from at least three independent cultures. All analyses were performed in a blinded manner with respect to the experimental groups.

### 2.7 Surface Biotinylation Assay

Astrocytes derived from CTRL, LRRK2‐PD‐1, and LRRK2‐PD‐2, as well as the corresponding gene‐corrected LRRK2‐ISO‐1 and LRRK2‐ISO‐2 iPSC lines, were plated in 6‐well plates at Day 70 and cultured in Advanced DMEM/F12 supplemented with 1% B27 and 1% GlutaMAX, without CNTF, until reaching confluency. Surface biotinylation was performed as previously described (Roh et al. 2025). Briefly, cells were washed once with ice‐cold PBS and incubated with 0.5 mg/mL sulfo‐NHS‐biotin for 30 min at 4°C in the dark with gentle agitation. The biotinylation reaction was then quenched by incubating the cells for 10 min at 4°C with a quenching buffer (1% BSA in PBS); then, cells were washed twice with ice‐cold PBS. Cells were lysed in 0.3 mL of lysis buffer containing 150 mM NaCl (Sigma Aldrich, cat. n° S9888), 50 mM Tris–HCl (pH 7.4) (Sigma Aldrich, cat. n° T1503), 1 mM EDTA (Promega, cat. n° V4233), 1% Triton X‐100, and protease inhibitor cocktail (Roche Diagnostics, Mannheim, Germany, cat. n° 11 697 498 001) and collected using cell scrapers. Lysates were centrifuged at 16000×*g* for 15 min at 4°C.

Protein concentration was determined using a DC protein assay (Bio‐Rad, Hercules, CA, USA, cat. n° 5 000111), and equal amounts of protein (200 μg) were incubated overnight at 4°C with streptavidin–agarose beads on an end‐over‐end rotator. Beads were then washed three times with lysis buffer, and bound proteins were eluted in sample buffer, containing 240 mM Tris–HCl (pH 6.8), 25% glycerol (Promega, cat. n° H5066), 12% sodium dodecyl sulfate (SDS; Sigma Aldrich, cat. n° L3771), 20% β‐mercaptoethanol (Sigma Aldrich, cat. n° M6250), and 0.04% bromophenol blue (Biorad, cat. n° 161‐0404) and incubated at 37°C for 1 h. Eluted proteins were subsequently resolved by SDS‐PAGE and analyzed by western blot as described below.

### 2.8 Western Blot Analyses

Cultures were washed with ice‐cold PBS and lysed in 50 mM Tris (pH 7.4) containing 150 mM NaCl, 0.5% sodium deoxycholate (Sigma Aldrich, cat. n° D6750), 0.1% SDS, 1% Igepal (Sigma Aldrich, cat. n° I8896), 1 mM polymethanesulphonyl fluoride (Sigma Aldrich, cat. n° 178 950), and a complete set of protease inhibitors.

Protein concentration was measured with a DC protein assay. Equal amounts of proteins (50 μg) were eluted in sample buffer, resolved on 10% SDS‐polyacrylamide gels and transferred onto PVDF membranes (Immobilon‐P; Millipore, cat. n° IPVH00010) using the TurboBlot system (Bio Rad).

Membranes were blocked with 5% non‐fat milk (GeneSpin, cat. n° STS‐M500) in 0.1 M Tris‐buffered saline (pH 7.4) containing 0.1% Tween‐20 (TBS‐T) for 30 min and then incubated overnight with the following primary antibodies: anti‐phospho‐Ser935 LRRK2 (1:1000), anti‐LRRK2 (1:10000), anti‐phospho‐Ser106‐Rab12 (1:1000), anti‐Rab12 (1:1000), anti‐GFAP (1:5000), anti‐D2R (1:100), or anti‐GAPDH (1:10000) (Table S2).

After primary antibody incubation, membranes were washed in TBS‐T and incubated with appropriate horseradish peroxidase–conjugated secondary antibodies (Santa Cruz Biotechnology, cat. n° sc‐2004, sc‐2031). Signals were detected by enhanced chemiluminescence (ECL) (GeneSpin, cat. n° STS‐E500). Blots were analyzed with ImageJ software. Protein levels were normalized to GAPDH as an internal loading control.

### 2.9 RNA Isolation and Gene Expression Analysis

Total RNA was extracted from astrocytes derived from CTRL, LRRK2‐PD‐1, and LRRK2‐PD‐2 and the relative gene‐corrected LRRK2‐ISO‐1 and LRRK2‐ISO‐2 iPSC lines at different time points of the differentiation process using TriZol Reagent (Life Technologies, cat. n° 15 596 026). RNA was quantified using the My Spect spectrophotometer (Biomed). For each sample, 1 μg of total RNA was reverse‐transcribed using the High‐Capacity cDNA Reverse Transcription kit from Life Technologies (cat. n° 4 368 814) using a Thermo cycler (Biorad) at 25°C for 10 min, 37°C for 120 min, and 85°C for 5 min. Quantitative RT‐PCR (qPCR) was performed using SybrGreen Master Mix (Bio‐Rad, cat. n° 1 725 270) and 20 ng of cDNA, in triplicate for each gene, and run on the ViiA7 instrument (Applied Biosystems). The following conditions were used: 95°C for 10 min for 1 cycle, 95°C for 15 s and 60°C for 1 min for 40 cycles. Expression levels of target genes were normalized on transcript levels of glyceraldehyde 3‐phosphate dehydrogenase (GAPDH). Primer sequences are listed in Table S4.

### 2.10 Calcium Imaging Analyses

Astrocytes derived from CTRL, LRRK2‐PD‐1, and LRRK2‐PD‐2, and the relative gene‐corrected LRRK2‐ISO‐1 and LRRK2‐ISO‐2 iPSC lines iPSC‐derived astrocytes (Day 70) were plated in a 4 well chamber slide (Ibidi, cat. n° 80 421) and incubated with Fluo4‐AM fluorophore (2.5 μM, Molecular Probes, cat. n° 11 504 786) for 30 min, slowly shaking at RT. Cells were then imaged during 5 min using a Zeiss LSM 880 confocal microscope equipped with Plan‐Apochromat 63X/1.4 numerical aperture oil objective, and recorded data were examined using Zen 2.3 Software (Carl Zeiss AG).

### 2.11 Growth Factors and Cytokine Analysis

Astrocytes derived from CTRL, LRRK2‐PD‐1, and LRRK2‐PD‐2 and the relative gene‐corrected LRRK2‐ISO‐1 and LRRK2‐ISO‐2 iPSC lines (day 70) were cultured for 14 days and supernatants collected and stored at −80°C. Quantification of the BDNF and NGF concentration in supernatants was performed using the Human BDNF ELISA Kit (ELK Biotechnology, cat n° ELK10262) and the Human NGF ELISA Kit (ELK Biotechnology, cat n° ELK1173) following the manufacturer's instructions. IL‐6 and IL‐8 production was measured using specific Duo‐Set kits (R&D system, cat. n° D6050B, D8000C) according to the manufacturer's protocol. Signal detection was performed using the Emax Plus, Microplate reader (Molecular Device).

### 2.12 Statistical Analyses

Experiments were independently conducted for each patient line (LRRK2‐PD: *n* = 2 LRRK2‐ISO: *n* = 2; CTRL: *n* = 1) and repeated at least three times. The normality tests were performed using the Shapiro–Wilk test. No statistical testing for outliers was performed. Significant differences from control conditions were determined using analysis of variance (ANOVA) followed by Bonferroni's test for multiple comparisons. Values are expressed as the mean ± standard error of the mean (SEM), unless otherwise stated. All statistical analyses were performed using GraphPad Prism version 4.00 for Windows (GraphPad Software, San Diego, CA, USA). All statistical results are shown in Tables S5–S14.

## 3 Results

### 3.1 Differentiation and Characterization of iPSC‐Derived Human Astrocytes

**FIGURE 1:**
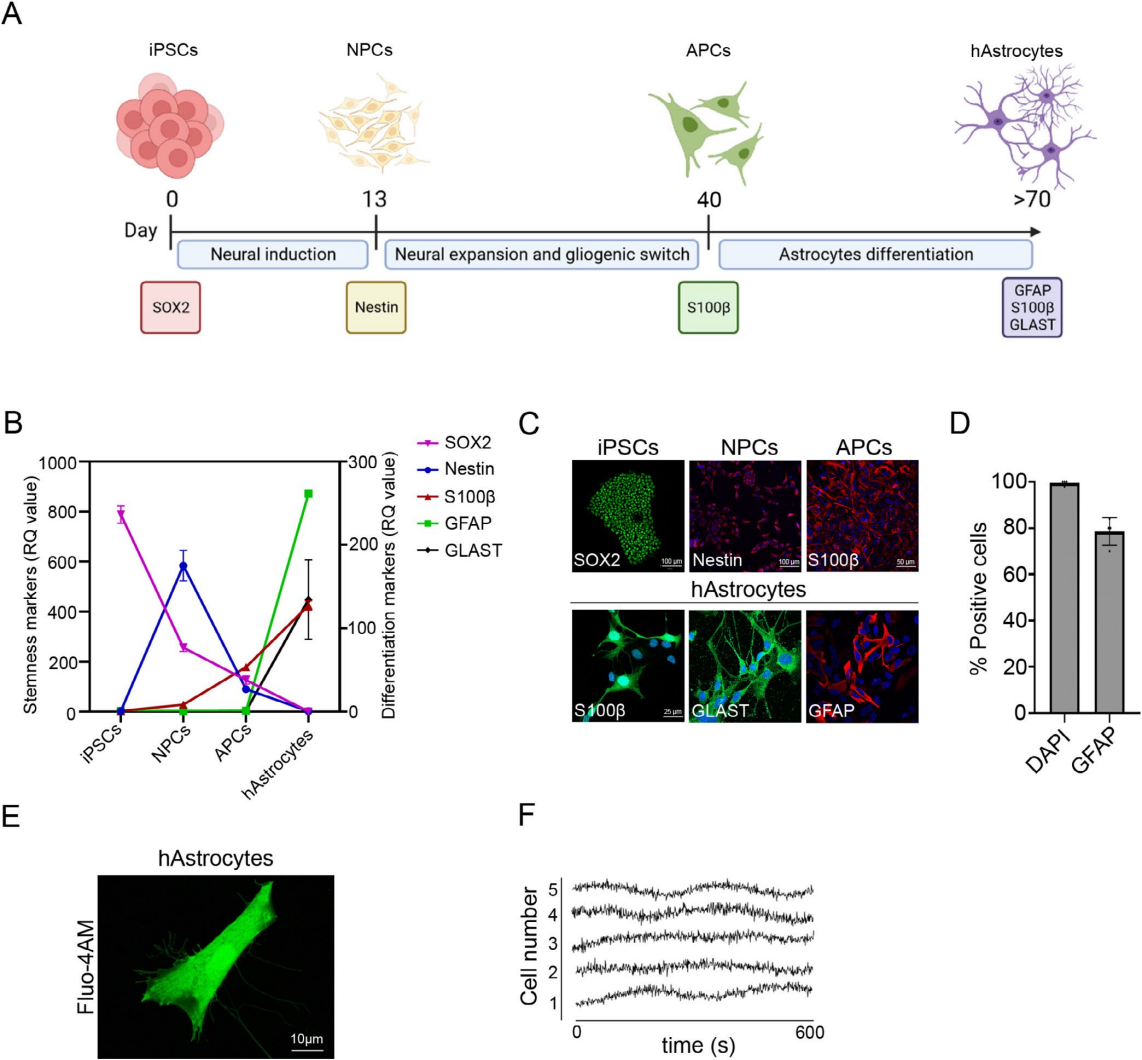
Characterization of human astrocytes differentiated from a control iPSC line. (A) Schematic timeline of differentiation of human induced pluripotent stem cells (iPSCs) from a healthy control into astrocytes (hAstrocytes). (B) Temporal expression of mRNA encoding SOX2, Nestin, S100β, GFAP, and GLAST, analyzed by qPCR in iPSCs (Day 0), neural progenitor cells (NPCs; day 13), astrocyte precursor cells (APCs; Day 40), and astrocytes (hAstrocytes; Day 70). Gene expression relative to GAPDH was assessed using the ΔCT method (RQ value) and was normalized to hAstrocytes (for SOX2) or to iPSCs (for Nestin, S100β, GFAP, and GLAST) which was arbitrarily set to a value of 1. Data are presented as mean values ± SEM. *n* = 3, *n* = number of independent cell culture preparations. (C) Representative immunofluorescence analysis of stage‐specific markers in iPSCs, NPCs, APCs, and hAstrocytes. Nuclei were stained with DAPI. (D) Quantification of the percentage of GFAP‐positive cells relative to the total cell population, identified by DAPI nuclear staining. Data are presented as mean values ± SEM. *n* = 3, *n* = number of independent cell culture preparations; individual points represent the average of data from each single experiment. (E, F) Functional characterization of hAstrocytes using the green fluorescent cell‐permeable calcium indicator, Fluo‐4 AM. (E) Representative cell labeled with Fluo‐4 AM and (F) spontaneous calcium wave recording for 300 s (s) (*n* = 5).

We generated astrocyte cultures from a human iPSC line derived from a healthy subject (Reinhardt et al. 2013) using an established protocol (Perriot et al. 2021) with minor modifications. Briefly, using a dual‐SMAD inhibition protocol (Chambers et al. 2009), iPSCs were cultured in the presence of the fibroblast growth factor 2 (FGF2) and the epidermal growth factor (EGF) until Day 13 to obtain neural progenitor cells (NPCs) (Sun et al. 2008). NPCs were then differentiated toward a proliferative population of astrocyte precursor cells (APCs) by culturing with the leukemia inhibitory factor (LIF) in combination with EGF (Day 40) (Bonni et al. 1997; Rowitch and Kriegstein 2010). APCs were cultured for an additional 4 weeks in the presence of the ciliary neurotrophic factor (CNTF), to obtain a population of non‐proliferating astrocytes (Day 70) (Figure 1A). Using quantitative real‐time polymerase chain reaction (qPCR) and immunofluorescence, the expression of canonical markers was analyzed throughout the differentiation process (Figure 1B,C). The mRNA encoding the SOX2 gene, involved in stem cell maintenance, was high on day zero, whereas its expression decreased during the differentiation process. The expression of the mRNA encoding Nestin, required for NPCs induction, was transiently and robustly induced around Day 13. mRNAs encoding genes typical of astrocytes, such as the S100 calcium binding protein beta (S100β), as well as the glial fibrillary acidic protein (GFAP) and the glutamate aspartate transporter (GLAST), which were induced starting from Days 13 and 40, respectively, peaked at Day 70. At the end of the differentiation protocol, the mRNA encoding neuronal markers, such as the pan neuronal microtubule‐associated protein 2 (MAP2) marker as well as the tyrosine hydroxylase (TH), here used as a marker for DA neurons, were totally undetectable (data not shown). The expression of SOX2, Nestin, S100β, GLATS and GFAP was also detected in the majority of cells in immunofluorescence experiments performed at Day 0, 13, 40 and 70 of the differentiation protocol from iPSCs to astrocytes (Figure 1C). In particular, at Day 70, ~80% of cells were positive for GFAP, used as a marker of astrocytes in subsequent experiments (Figure 1D). Spontaneous intracellular calcium waves have been used as a measure of astrocytes functionality (Di Domenico et al. 2019; Filippini et al. 2021). iPSC‐derived human astrocytes (Day 70) were incubated with Fluo‐4 AM, a green fluorescent cell‐permeable indicator able to monitor astrocytic calcium fluctuations at rest conditions and recorded for 5 min using confocal microscopy. As shown in Figure 1E,F, iPSC‐derived cultures displayed spontaneous calcium spikes that, along with previous observations, indicate the capability to generate a nearly pure population of functional astrocytes.

### 3.2 D2R in iPSC‐Derived Human Astrocytes Promotes Mouse DA Neuron Neurotrophic Effects

**FIGURE 2:**
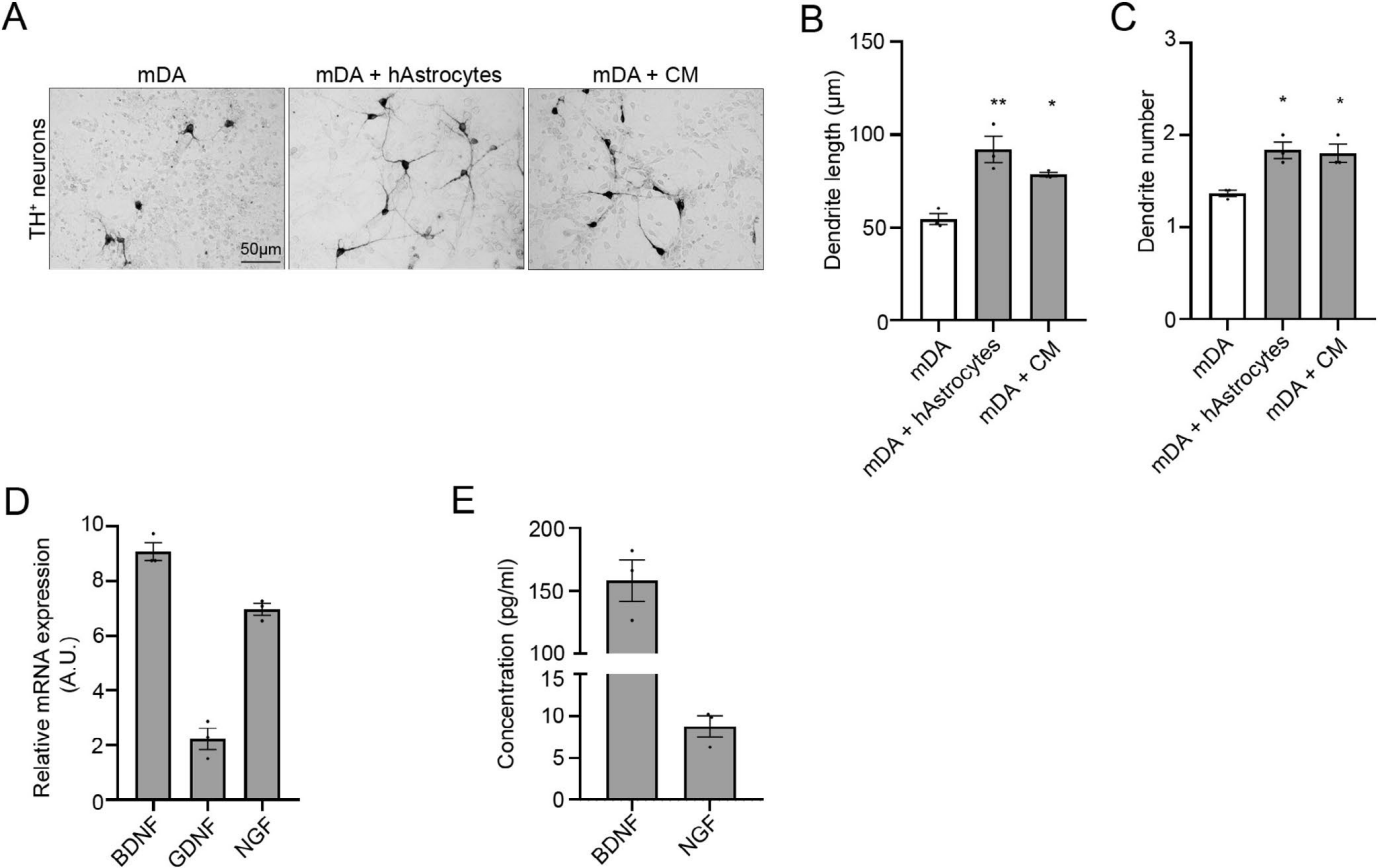
Functional characterization of human astrocytes differentiated from a control iPSC line. (A) Representative images of mouse primary dopaminergic neurons (mDA) stained with anti‐tyrosine hydroxylase (TH) primary antibody, cultured alone, co‐cultured with iPSC‐derived astrocytes (Day 70) (mDA + hAstrocytes), or exposed to iPSC‐derived astrocyte‐conditioned medium (mDA + CM). (B, C) Morphological analysis of the length of the primary dendrite (B) and dendrite number (C) of mDA neurons under different culture conditions. Data are presented as mean values ± SEM. *n* = 3, *n* = number of independent cell culture preparations; individual points represent the average of data from each single experiment (**p < 0.01, *p < 0.05 vs. mDA; one‐way ANOVA followed by post hoc Bonferroni's test). (D) Relative mRNA expression of genes encoding the brain derived neurotrophic factor (BDNF), the glial cell line‐derived neurotrophic factor (GDNF) and the nerve growth factor (NGF) in iPSC‐derived astrocytes (Day 70), measured by qPCR and normalized to GAPDH. (E) BDNF and NGF levels released by iPSC‐derived astrocytes (Day 70), measured by ELISA assay. Data are presented as mean values ± SEM. *n* = 3, *n* = number of independent cell culture preparations; individual points represent the average of data from each single experiment.

It is well known that astrocytes secrete neurotrophic factors, such as brain‐derived neurotrophic factor (BDNF), nerve growth factor (NGF), and glial cell line‐derived neurotrophic factor (GDNF), that ensure correct neuronal development, differentiation and survival (Miyazaki and Asanuma 2020). To investigate the ability of astrocytes in promoting DA neuron trophism, co‐cultures were developed by combining primary cultures of mouse midbrain neurons containing DA neurons (Bontempi et al. 2017; Mutti et al. 2022), and iPSC‐derived human astrocytes (Figure 2). Mixed species human–mouse co‐cultures are, in fact, widely used as experimental systems to investigate neuron–glia interactions, since multiple lines of evidence have clearly demonstrated a cross‐reactivity between neurotrophins and their receptors across mammalian species (Bono et al. 2018, 2021; Fenske et al. 2019; Filippini et al. 2021; Uchino et al. 2022). In particular, mouse midbrain neurons were seeded on a confluent monolayer of iPSC‐derived human astrocytes (Day 70) and cultured for 7 days. In parallel experiments, mouse midbrain neurons were seeded and cultured for 7 days on a poly‐D‐lysine/laminin coating, used as a control. Cultures were then analyzed for mouse DA neuron morphology in immunocytochemistry experiments and computer‐assisted morphometry. The anti‐TH primary antibody was used to detect DA neurons. As key morphological endpoints, we examined mouse DA neuron maximal length of the primary dendrite and dendrite numbers (Bontempi et al. 2017; Bono et al. 2018; Mutti et al. 2022). We found that TH‐positive mouse DA neurons co‐cultured with human astrocytes exhibited a significant increase in both primary dendrite length (Figure 2A,B) and dendrite numbers (Figure 2A,C) compared to neurons cultured without astrocytic support. Moreover, mouse DA neurons seeded on poly‐D‐lysine/laminin coating were cultured for 7 days in the presence of medium conditioned by iPSC‐derived astrocytes (70 day) and morphologically analyzed. The results show that the increase in both primary dendrite length and dendrite numbers did not differ from that observed in co‐culture experiments (Figure 2A–C). Therefore, astrocyte‐secreted factors are crucial for promoting neuronal morphological effects. Accordingly, qPCR analysis of mRNA from iPSC‐derived astrocytic cultures (Day 70 of differentiation) revealed elevated expression of growth factor genes, mainly BDNF and NGF, but also GDNF, albeit to a lesser extent (Figure 2D). Consistent with these results, high levels of released BDNF and NGF in the conditioned media of iPSC‐derived astrocytes were measured by ELISA (Figure 2E).

**FIGURE 3:**
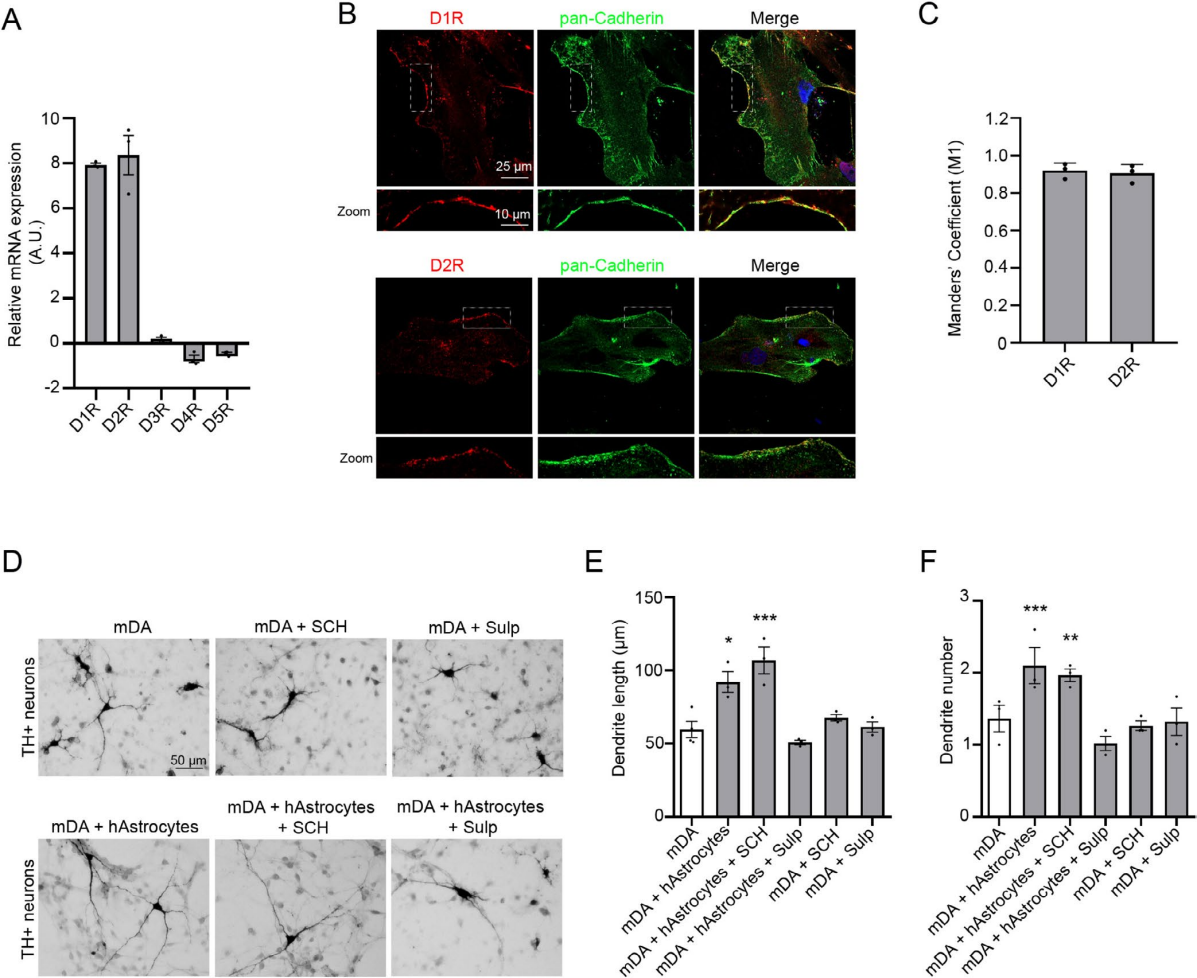
D2R expression in human astrocytes differentiated from a control iPSC line. (A) Relative mRNA expression of genes encoding DA D1 receptor (D1R), D2 receptor (D2R), D3 receptor (D3R), D4 receptor (D4R) and D5 receptor (D5R) in iPSC‐derived astrocytes (Day 70) from a healthy control, measured by qPCR and normalized to GAPDH. Data are presented as mean values ± SEM. *n* = 3, *n* = number of independent cell culture preparations; individual points represent the average of data from each single experiment. (B) Representative co‐immunofluorescence images showing D1R (top) and D2R (bottom) (both in red) together with the plasma membrane marker pan‐cadherin (in green) in iPSC‐derived astrocytes (Day 70). Nuclei were stained with DAPI (blue). (C) Quantification of D1R and cadherin colocalization, or D2R and cadherin colocalization in iPSC‐derived astrocytes (Day 70) performed using the Manders' overlap coefficient. Values range from 0 (no colocalization) to 1 (complete colocalization). Data are presented as mean values ± SEM. *n* = 3, *n* = number of independent cell culture preparations; individual points represent the average of data from each single experiment. (D) Representative images of mDA neurons stained with TH, cultured alone (top) or co‐cultured with iPSC‐hAstrocytes (bottom), both exposed to the D1R antagonist SCH23990 (SCH; 10 μM) or the D2R antagonist sulpiride (Sulp; 5 μM) for 5 days. (E, F) Morphological analysis of the length of the primary dendrite (E) and dendrite number (F) of mDA neurons under different culture conditions. Data are presented as mean values ± SEM. *n* = 3, *n* = number of independent cell culture preparations; individual points represent the average of data from each single experiment (***p < 0.001, **p < 0.01, *p < 0.05 vs. mDA; one‐way ANOVA followed by post hoc Bonferroni's test).

Since receptors for DA have been clearly associated with the ability of astrocytes in secreting neurotrophic mediators (Broome Thomas et al. 2020; Miyazaki and Asanuma 2020). iPSC‐derived cultures of human astrocytes were analyzed for the mRNAs encoding both D1‐like (D1R and D5R) and D2‐like (D2R, D3R and D4R) receptors by qPCR. As shown in Figure 3, human astrocytes expressed high levels of D1R and D2R, while D3R, D4R, and D5R mRNAs were almost undetectable (Figure 3A). Then, localization of D1R and D2R at the plasma membrane was analyzed by immunofluorescence in co‐localisation experiments, using specific anti‐D1R and anti‐D2R primary antibodies together with an antibody anti‐pan‐cadherin used as a marker for plasma membrane (Teo et al. 2019). As shown in Figure 3B, a high level of overlap between cadherins and D1R or D2R has been measured, thus suggesting that receptors are located at the plasma membrane (Figure 3C).

To investigate the role of D1R and D2R in promoting neuronal trophism, mouse DA neurons/iPSC‐derived astrocyte co‐cultures or poly‐D‐lysine/laminin‐coated mouse DA neuron cultures were incubated with either SCH23990, a D1R antagonist (10 μM) or sulpiride, a D2R antagonist (5 μM) for 5 days. Mouse DA neurons were then morphologically analyzed by immunocytochemistry. As shown in Figure 3, in TH‐positive neurons co‐cultured with astrocytes, the improvement in both the primary dendrite length and dendrite numbers was significantly counteracted by sulpiride, but not by SCH23990, clearly pointing to the astrocyte D2R as a key receptor in promoting DA neuron remodeling likely increasing the release of neurotrophic mediators (Figure 3D–F). Moreover, the contribution of neuronal D1R and D2R was excluded by the observation that, in control mouse DA neurons neither SCH23990 nor sulpiride were able to affect TH‐positive neuron morphology (Figure 3D–F).

### 3.3 G2019S Mutation in LRRK2 Affects iPSC‐Derived Astrocytes Activity and D2R Cell Membrane Expression

LRRK2 is highly expressed in astrocytes, and G2019S mutation has been shown to affect astrocyte activity, leading to reactive astrocytes (Bailey and Cookson 2024). Upregulation of GFAP and hypertrophy has usually been described as prominent features of reactive astrocytes (Escartin et al. 2021; Booth et al. 2017; Miyazaki and Asanuma 2020). In this study, human astrocytes were obtained by differentiating iPSC generated from two PD patients harboring the G2019S LRRK2 mutation (LRRK2‐PD‐1 and LRRK2‐PD‐2), and their respective G2019S LRRK2 isogenic iPSC lines with the correction of the mutation (LRRK2‐ISO‐1 and LRRK2‐ISO‐2) and from a healthy control (CTRL) (Table S1) (Reinhardt et al. 2013), as described above. Analyses of phosphorylation of LRRK2 at the serine 935 (pSer935 LRRK2), the active form of the enzyme, and its specific substrate Rab12, at the serine 106 (pSer106 Rab12), were performed by western blot. Figure S1 shows that, in contrast to LRRK2‐ISO‐1 and LRRK2‐ISO‐2, as well as to healthy control‐derived astrocytes, in LRRK2‐PD‐1 and LRRK2‐PD‐2‐derived astrocytes both ser935 LRRK2 and ser106 Rab12 were highly phosphorylated.

**FIGURE 4:**
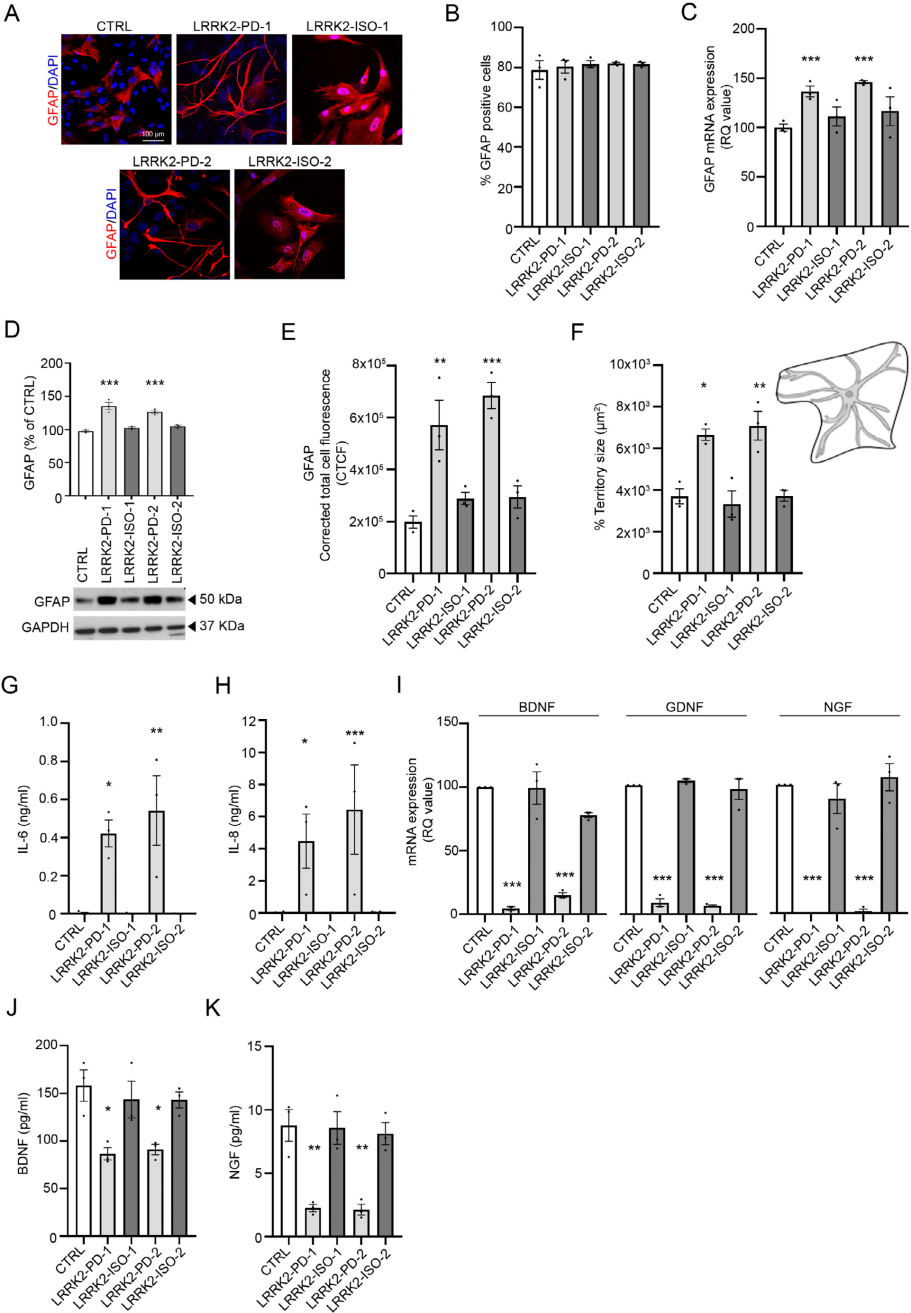
Characterization of human astrocytes differentiated from two iPSC lines of PD patients carrying the LRRK2 G2019S mutation. (A) Representative immunofluorescence images showing GFAP (red) expression in iPSC‐derived astrocytes (Day 70) obtained from a healthy control (CTRL), two LRRK2‐PD patients (LRRK2‐PD‐1 and LRRK2‐PD‐2), and their respective isogenic lines (LRRK2‐ISO‐1 and LRRK2‐ISO‐2). Nuclei were stained with DAPI (blue). (B) Quantification of the percentage of GFAP‐positive cells relative to the total cell population, identified by DAPI nuclear staining, in CTRL‐, LRRK2‐PD‐ and LRRK2‐ISO‐derived cultures. Data are presented as mean values ± SEM. *n* = 3, *n* = number of independent cell culture preparations; individual points represent the average of data from each single experiment. (C) Quantitative PCR analysis of GFAP mRNA expression in iPSC‐derived astrocytes from CTRL, LRRK2‐PD and LRRK2‐ISO lines. Gene expression relative to GAPDH was assessed using the ΔCT method (RQ value) and was normalized to CTRL, which was arbitrarily set to a value of “100”. Data are presented as mean values ± SEM. *n* = 3, *n* = number of independent cell culture preparations; individual points represent the average of data from each single experiment (***p < 0.001 vs. CTRL; one‐way ANOVA followed by Bonferroni's post hoc test). (D) Densitometric analysis of Western blots with specific levels of GFAP normalized to GAPDH levels (top). Representative image of blots of GFAP and GAPDH in iPSC‐derived astrocytes from CTRL, LRRK2‐PD and LRRK2‐ISO lines (bottom). Data are presented as mean values ± SEM. *n* = 3, *n* = number of independent cell culture preparations; individual points represent the average of data from each single experiment (***p < 0.001 vs. CTRL; one‐way ANOVA followed by Bonferroni's post hoc test). (E) Corrected total cell fluorescence intensity for GFAP expression and (F) quantification of cell territory size of iPSC‐derived astrocytes obtained from CTRL, LRRK2‐PD and LRRK2‐ISO lines. Data are presented as mean values ± SEM. *n* = 3, *n* = number of independent cell culture preparations; individual points represent the average of data from each single experiment (***p < 0.001, **p < 0.01, *p < 0.05 vs. CTRL; one‐way ANOVA followed by Bonferroni's post hoc test). (G‐H) Levels of IL‐6 (G) and IL‐8 (H) in the culture medium of iPSC‐derived astrocytes from CTRL, LRRK2‐PD and LRRK2‐ISO lines, measured by ELISA. Data are presented as mean values ± SEM. *n* = 3, *n* = number of independent cell culture preparations; individual points represent the average of data from each single experiment (***p < 0.001, **p < 0.01, *p < 0.05 vs. CTRL; one‐way ANOVA followed by Bonferroni's post hoc test). (I) Quantitative PCR analysis of genes encoding BDNF, GDNF and NGF in iPSC‐derived astrocytes from CTRL, LRRK2‐PD and LRRK2‐ISO lines. Gene expression relative to GAPDH was assessed using the ΔCT method (RQ value) and was normalized to CTRL, which was arbitrarily set to a value of “100”. Data are presented as mean values ± SEM. *n* = 3, *n* = number of independent cell culture preparations; individual points represent the average of data from each single experiment (***p < 0.001 vs. CTRL; one‐way ANOVA followed by Bonferroni's post hoc test). (J, K) Levels of BDNF (J) and NGF (K) released in the culture medium of iPSC‐derived astrocytes (Day 70) from CTRL, LRRK2‐PD and LRRK2‐ISO lines, measured by ELISA. Data are presented as mean values ± SEM. *n* = 3, *n* = number of independent cell culture preparations; individual points represent the average of data from each single experiment (**p < 0.01, *p < 0.05 vs. CTRL; one‐way ANOVA followed by Bonferroni's post hoc test).

Cultures derived from LRRK2‐PD‐1 and LRRK2‐PD‐2 were then analyzed in immunofluorescence experiments, showing that around 80% of cells were positive for the GFAP astrocytes marker, a percentage that did not differ from those observed in CTRL cultures and in cultures derived from LRRK2‐ISO‐1 and LRRK2‐ISO‐2 in which the mutation was corrected (Figure 4A,B). Therefore, at the time point analyzed, G2019S mutation in LRRK2 did not change the amount of GFAP‐positive cells. Moreover, intracellular calcium waves were analyzed using the Fluo‐4 AM fluorophore, showing spontaneous and transient calcium signals in all the iPSC‐derived astrocytes, as observed in CTRL experiments (Figure S2A,B). At day 70, the mRNAs encoding S100β, GLAST, and GFAP were measured using qPCR, showing that LRRK2‐PD‐1 and LRRK2‐PD‐2 cultures were characterized by a significant increase in the expression levels of mRNA for GFAP (Figure 4C), but not of S100β and GLAST (Figure S2C,D) if compared to those measured in astrocytes derived from their respective LRRK2‐ISO‐1 and LRRK2‐ISO‐2 and CTRL iPSC lines. Thus, there is an association between G2019S LRRK2 mutation and GFAP mRNA upregulation. GFAP upregulation was further investigated by Western blot showing a significant increase in GFAP protein levels in LRRK2‐PD‐1 and LRRK2‐PD‐2 astrocytes compared to LRRK2‐ISO‐1, LRRK2‐ISO‐2, and CTRL iPSC‐derived astrocytes (Figure 4D). Consistently, immunofluorescence analysis showed a significantly higher GFAP staining intensity in LRRK2‐PD astrocytes (Figure 4E).

Moreover, astrocyte hypertrophy was quantified by measuring the total area covered by each cell (Zhang et al. 2016), showing that LRRK2‐PD‐1 and LRRK‐PD‐2 astrocytes displayed a larger territorial size compared to astrocytes derived from LRRK2‐ISO and CTRL iPSC (Figure 4F). Since reactive astrocytes are characterized by their ability to secrete various cytokines, culture media from LRRK2‐PD‐1 and LRRK2‐PD‐2 were analyzed by ELISA for IL‐6 and IL‐8 levels, two cytokines involved in PD neuroinflammation (Brockmann et al. 2017; Morimoto et al. 2022; Pons‐Espinal et al. 2024), and compared to those measured in LRRK2‐ISO‐1, LRRK2‐ISO‐2 and CTRL astrocyte culture media (Day 70). As shown in Figure 4G,H, IL‐6 and IL‐8 were highly detected in the media derived from LRRK2‐PD‐1 and LRRK2‐PD‐2, but not in those derived from LRRK2‐ISO‐1 and LRRK2‐ISO‐2 and CTRL astrocytes. Intriguingly, the mRNA encoding BDNF, GDNF and NGF was detected at very low levels in LRRK2‐PD‐1 and LRRK2‐PD‐2 cultures when compared to astrocytes derived from LRRK2‐ISO‐1, LRRK2‐ISO‐2 and CTRL iPSC (Figure 4I). These findings were further confirmed by ELISA analysis, showing reduced levels of released BDNF and NGF in the culture media of LRRK2‐PD astrocytes (Figure 4J,K, respectively). In line with previous reports, these data indicate that astrocytes carrying G2019S LRRK2 mutation lose their neurotrophic properties and exhibit morphological, molecular, and functional features typical of a reactive phenotype.

**FIGURE 5:**
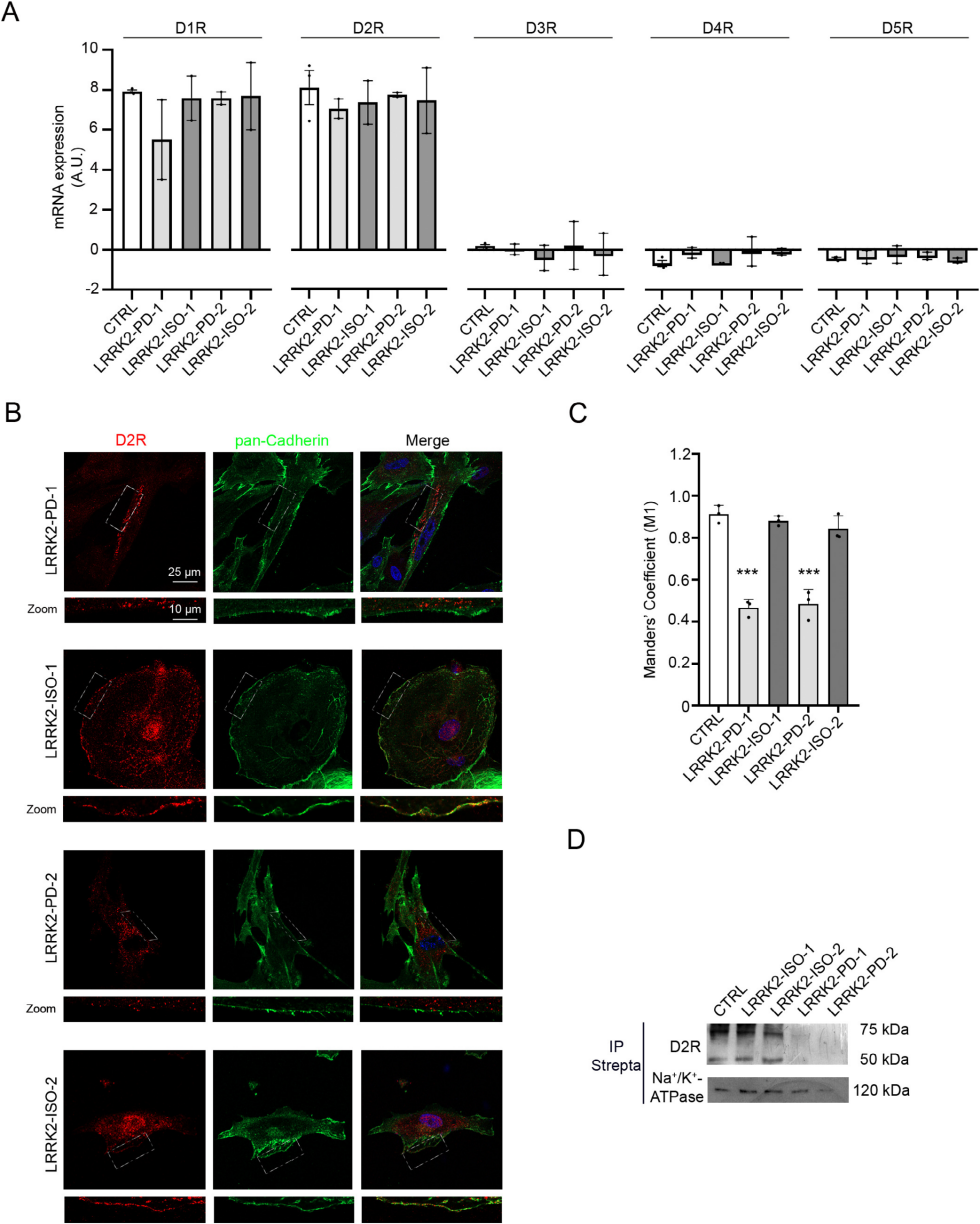
Analyses of DA receptor expression in astrocytes derived from LRRK2‐PD iPSC lines. (A) Relative mRNA expression of genes encoding D1R, D2R, D3R, D4R, and D5R in iPSC‐derived astrocytes from CTRL, LRRK2‐PD and LRRK2‐ISO lines (Day 70) measured by qPCR and normalized to GAPDH. Data are presented as mean values ± SEM. *n* = 3, *n* = number of independent cell culture preparations; individual points represent the average of data from each single experiment. (B) Representative immunofluorescence images showing D2R (red) and pan‐cadherin (green) colocalization in iPSC‐derived astrocytes from LRRK2‐PD and LRRK2‐ISO lines. Nuclei were stained with DAPI (blue). (C) Quantification of D2R and cadherin colocalization in iPSC‐derived astrocytes from CTRL, LRRK2‐PD, and LRRK2‐ISO lines (Day 70) performed using the Manders' overlap coefficient. Values range from 0 (no colocalization) to 1 (complete colocalization). Data are presented as mean values ± SEM. *n* = 3, *n* = number of independent cell culture preparations; individual points represent the average of data from each single experiment (***p < 0.001 vs. CTRL; one‐way ANOVA followed by Bonferroni's post hoc test). (D) Analysis of cell‐surface D2R expression by biotinylation assay. Cell‐surface proteins were labeled with Sulfo‐NHS‐SS‐Biotin followed by lysis and immunoprecipitated for streptavidin. The isolated fraction was analyzed by Western blot using an anti‐D2R antibody. Na^+^/K^+^‐ATPase was used as a control. Representative blot is shown.

We, and others, previously described that the G2019S mutation impairs the membrane localization of several receptors (Bono et al. 2021; Migheli et al. 2013; Rassu et al. 2017). On these bases, we first investigated astrocyte DA receptor expression by using qPCR (Figure 5A). As shown for astrocytes derived from CTRL iPSC, those derived from both LRRK2‐PD‐1 and LRRK2‐PD‐2, as well as from LRRK2‐ISO‐1 and LRRK2‐ISO‐2, were characterized by high levels of mRNA encoding D1R and D2R, with superimposable results among the groups (Figure 5A). Next, given their crucial role in preserving DA neuron health, D2R localization was investigated by immunofluorescence in co‐localisation experiments, using anti‐D2R and anti‐pan‐cadherin primary antibodies (Figure 5B,C). The results report that while a high level of overlap between cadherins and D2R has been measured in CTRL iPSC‐derived astrocytes, as well as in LRRK2‐ISO‐1 and LRRK2‐ISO‐2 astrocytes, astrocytes derived from LRRK2‐PD‐1 and LRRK2‐PD‐2 iPSC lines showed a significantly reduced D2R‐cadherin co‐localization level (M1; LRRK2‐PD‐1: −45% ± 4.0% of reduction vs. CTRL; LRRK2‐PD‐2: −43% ± 6.9% of reduction vs. CTRL). Together, these data suggest that a reduced membrane expression of D2R is an additional molecular feature of astrocytes carrying the G2019S mutation in LRRK2. To further validate these findings, a cell surface biotinylation assay was performed in order to isolate plasma membrane‐associated proteins, followed by Western blot analysis for D2R. Na^+^/K^+^‐ATPase was used as a loading control (Roh et al. 2025). The result showed that in CTRL astrocytes, two D2R‐immunoreactive bands were detected, at approximately 50 and 75 kDa, corresponding to the canonical and glycosylated forms of the receptor, respectively (Fishburn et al. 1995) (Figure 5D). By contrast, in LRRK2‐PD‐1 astrocytes, the D2R signal was markedly reduced (% of reduction vs. control: 50 kDa: −99 ± 0.68; 75 kDa: −98 ± 0.44) and undetectable in LRRK2‐PD‐2 samples. In LRRK2‐ISO‐1 and LRRK2‐ISO‐2, the 50 and 75 kDa D2R levels were comparable to that measured in CTRL astrocytes (Figure 5D).

Overall, these results support an impairment in D2R membrane localization in PD‐derived astrocytes condition. This observation is relevant since activation of D2R, besides its role in inducing the secretion of neurotrophic mediators, has been associated with the suppression of neuroinflammation (Broome Thomas et al. 2020).

### 3.4 Normalizing G2019S LRRK2 Kinase Activity Restores Neurotrophic Properties of iPSC‐Derived Astrocytes and D2R Localization at the Plasma Membrane

**FIGURE 6:**
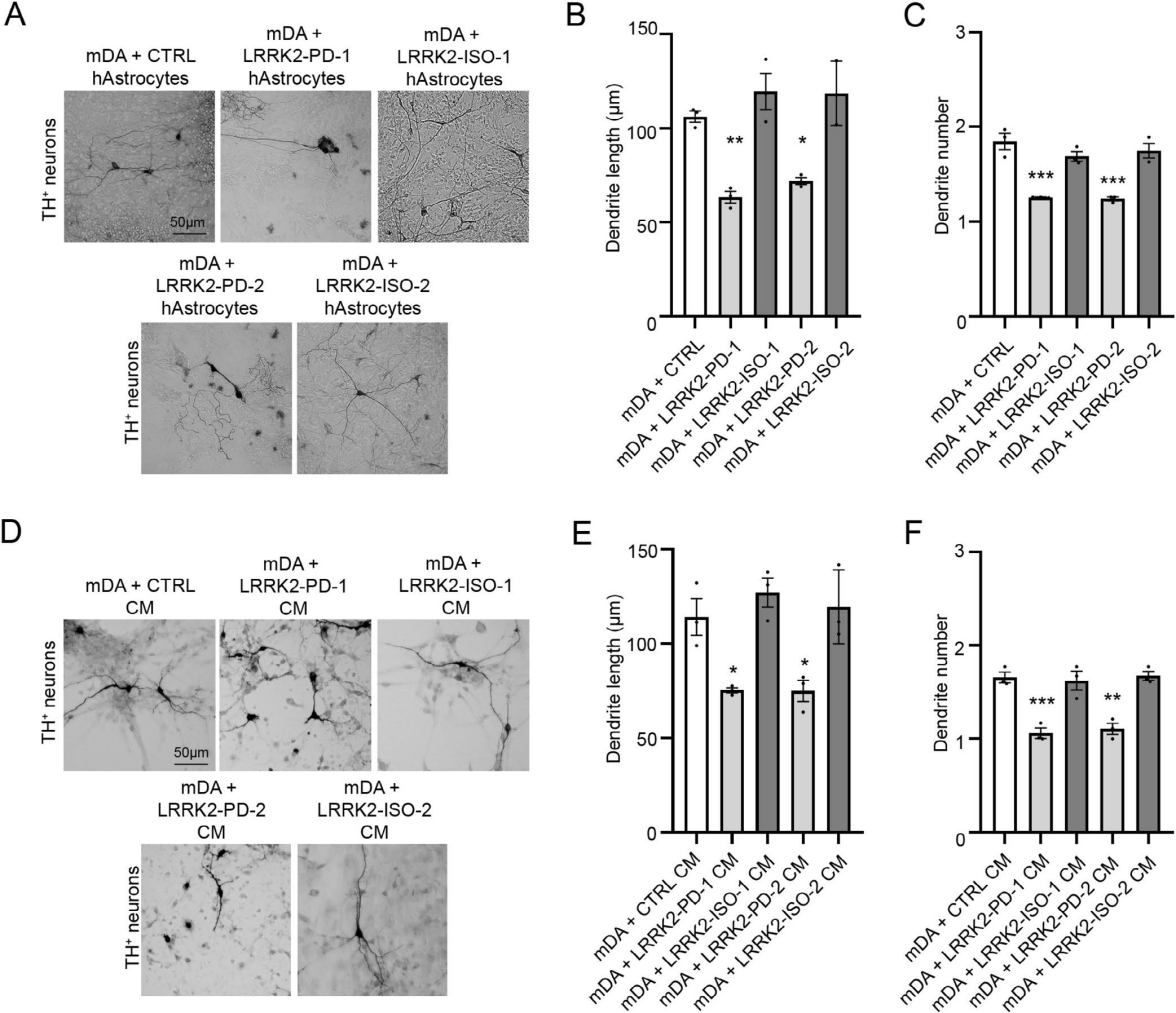
Functional characterization of human astrocytes differentiated from LRRK2‐PD iPSC lines. (A) Representative images of mDA neurons stained with TH, cultured alone or co‐cultured with hAstrocytes (Day 70) derived from CTRL, LRRK2‐PD and LRRK2‐ISO iPSC lines. (B‐C) Morphological analysis of primary dendrite length (B) and dendrite number (C) of mDA neurons under different culture conditions. Data are presented as mean values ± SEM. *n* = 3, *n* = number of independent cell culture preparations; individual points represent the average of data from each single experiment (***p < 0.001, **p < 0.01, *p < 0.05 vs. mDA; one‐way ANOVA followed by post hoc Bonferroni's test). (D) Representative images of mDA neurons cultured alone or exposed to the media conditioned (CM) by the astrocytes derived from CTRL, LRRK2‐PD and LRRK2‐ISO iPSC lines. (E‐F) Morphological analysis of primary dendrite length (E) and dendrite number (F) of mDA neurons under different culture conditions. Data are presented as mean values ± SEM. *n* = 3, *n* = number of independent cell culture preparations; individual points represent the average of data from each single experiment (***p < 0.001, **p < 0.01, *p < 0.05 vs. mDA; one‐way ANOVA followed by post hoc Bonferroni's test).

To examine the impact of G2019S LRRK2 mutation in astrocyte function, astrocytes derived from LRRK2‐PD‐1, LRRK‐PD‐2, LRRK2‐ISO‐1, LRRK‐ISO‐2 and CTRL iPSC (Day 70) were co‐cultured for 7 days with mouse DA neurons, and the TH‐positive neurons were morphologically analyzed. As shown in Figure 6, both the LRRK2‐PD‐derived cultures increased the primary dendrite length and dendrite numbers of TH‐positive neurons as observed in TH‐positive neurons co‐cultured with CTRL astrocytes and gene‐corrected LRRK2‐ISO‐1, LRRK2‐ISO‐2 astrocytes (Figure 6A–C).

Next, mouse DA neurons were exposed for 7 days to the media conditioned by iPSC‐derived LRRK2‐PD, LRRK2‐ISO, or CTRL cultures. TH‐positive neurons were then morphologically analyzed, showing that the increase in dendrite length and number induced by LRRK2‐ISO‐1, LRRK2‐ISO‐2, or CTRL conditioned media was not observed when mouse DA neurons were exposed to LRRK2‐PD‐1 and LRRK2‐PD‐2 conditioned media (Figure 6D–F).

**FIGURE 7:**
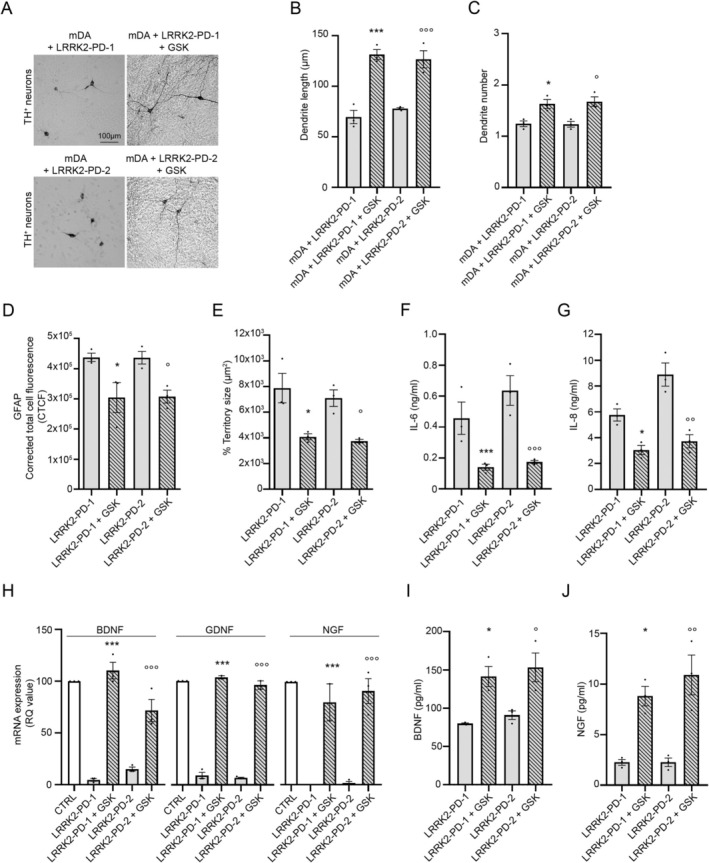
Effects of the LRRK2 kinase inhibitor GSK2578215A on LRRK2‐PD‐derived astrocytes function. Astrocytes derived from LRRK2‐PD iPSC (Day 70) were treated with the LRRK2 inhibitor GSK2578215A (GSK) for 10 days. (A) Representative images of mDA neurons stained with TH, cultured in the presence of untreated or GSK‐treated astrocytes derived from LRRK2‐PD iPSC lines. (B, C) Morphological analysis of primary dendrite length (B) and dendrite number (C) of mDA neurons under different culture conditions. Data are presented as mean values ± SEM. *n* = 3, *n* = number of independent cell culture preparations; individual points represent the average of data from each single experiment (***p < 0.001, *p < 0.05 vs. LRRK2‐PD‐1; °°°*p* < 0.001, °*p* < 0.05 vs. LRRK2‐PD‐2; one‐way ANOVA followed by post hoc Bonferroni's test). (D) Corrected total cell fluorescence intensity for GFAP expression and (E) quantification of astrocyte territory size of untreated or GSK‐treated astrocytes derived from LRRK2‐PD iPSC lines. Data are presented as mean values ± SEM. *n* = 3, *n* = number of independent cell culture preparations; individual points represent the average of data from each single experiment (*p < 0.05 vs. LRRK2‐PD‐1; °*p* < 0.05 vs. LRRK2‐PD‐2; one‐way ANOVA followed by Bonferroni's post hoc test). (F‐G) Levels of IL‐6 (F) and IL‐8 (G) in the culture medium of untreated or GSK‐treated astrocytes derived from LRRK2‐PD iPSC lines, measured by ELISA. Data are presented as mean values ± SEM. *n* = 3, *n* = number of independent cell culture preparations; individual points represent the average of data from each single experiment (***p < 0.001, *p < 0.05 vs. LRRK2‐PD‐1; °°°*p* < 0.001, °°*p* < 0.01 vs. LRRK2‐PD‐2; one‐way ANOVA followed by Bonferroni's post hoc test). (H) Quantitative PCR analysis of genes encoding BDNF, GDNF, and NGF in untreated or GSK‐treated astrocytes derived from LRRK2‐PD iPSC lines. Gene expression relative to GAPDH was assessed using the ΔCT method (RQ value) and was normalized to CTRL, which was arbitrarily set to a value of 100. Data are presented as mean values ± SEM. *n* = 3, *n* = number of independent cell culture preparations; individual points represent the average of data from each single experiment (***p < 0.001 vs. LRRK2‐PD‐1; °°°*p* < 0.001 vs. LRRK2‐PD‐2; one‐way ANOVA followed by Bonferroni's post hoc test). (I, J) Levels of BDNF (I) and NGF (J) released in the culture medium of iPSC‐derived astrocytes from untreated and GSK‐treated astrocytes derived from LRRK2‐PD iPSC lines, measured by ELISA. Data are presented as mean values ± SEM. *n* = 3, *n* = number of independent cell culture preparations; individual points represent the average of data from each single experiment (*p < 0.05 vs. LRRK2‐PD‐1; °°*p* < 0.01, °*p* < 0.05 vs. LRRK2‐PD‐2; one‐way ANOVA followed by Bonferroni's post hoc test).

**FIGURE 8:**
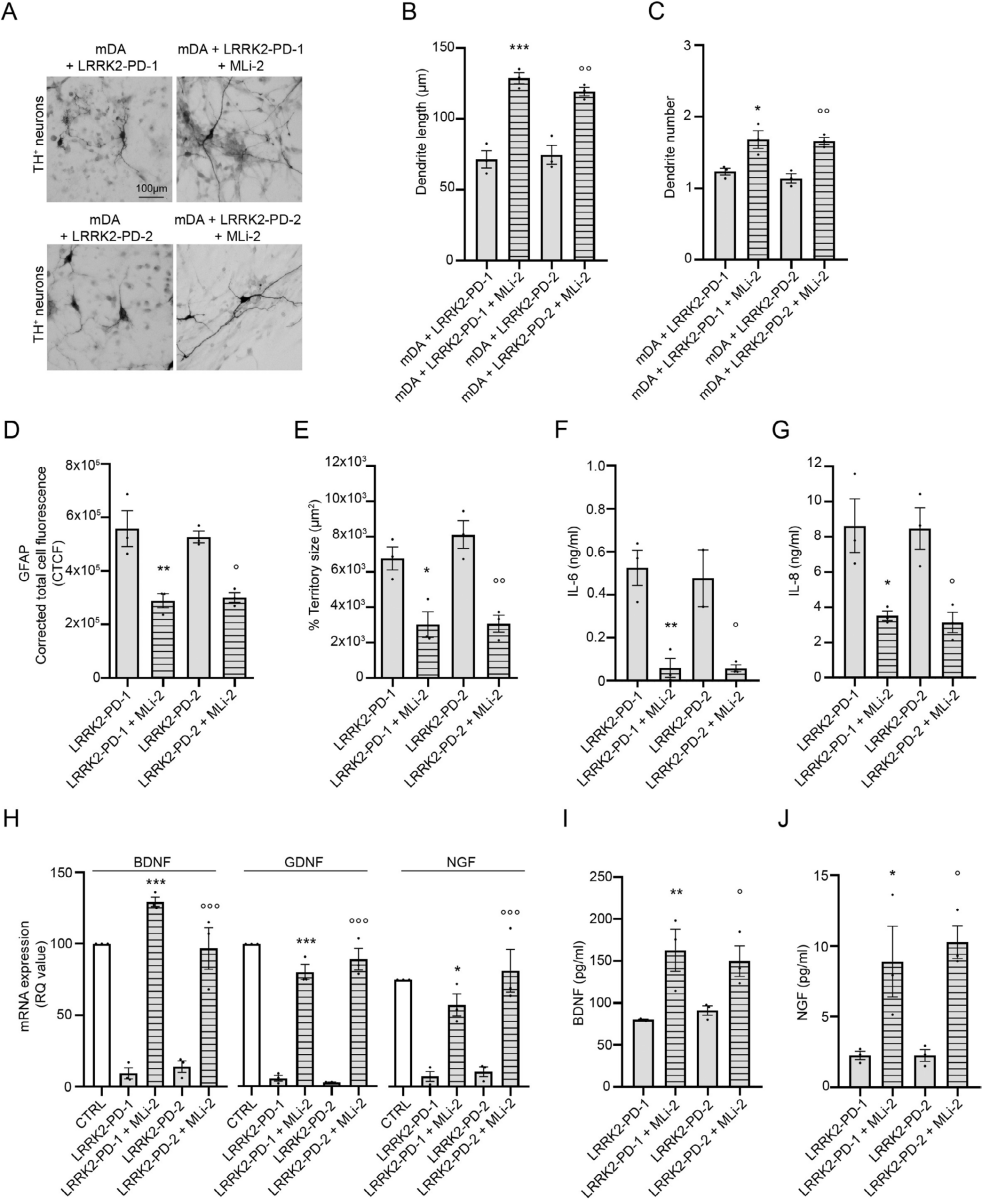
Effects of the LRRK2 kinase inhibitor MLi‐2 on LRRK2‐PD‐derived astrocytes function. Astrocytes derived from LRRK2‐PD iPSC (Day 70) were treated with the LRRK2 inhibitor MLi‐2 for 10 days. (A) Representative images of mDA neurons stained with TH, cultured in the presence of untreated or MLi‐2‐treated astrocytes derived from LRRK2‐PD iPSC lines. (B‐C) Morphological analysis of primary dendrite length (B) and dendrite number (C) of mDA neurons under different culture conditions. Data are presented as mean values ± SEM. *n* = 3, *n* = number of independent cell culture preparations; individual points represent the average of data from each single experiment (***p < 0.001, *p < 0.05 vs. LRRK2‐PD‐1; °°*p* < 0.01vs LRRK2‐PD‐2; one‐way ANOVA followed by post hoc Bonferroni's test). (D) Corrected total cell fluorescence intensity for GFAP expression and (E) quantification of astrocyte territory size of untreated or MLi‐2‐treated astrocytes derived from LRRK2‐PD iPSC lines. Data are presented as mean values ± SEM. *n* = 3, *n* = number of independent cell culture preparations; individual points represent the average of data from each single experiment (**p < 0.01, *p < 0.05 vs. LRRK2‐PD‐1; °°*p* < 0.01, °*p* < 0.05 vs. LRRK2‐PD‐2; one‐way ANOVA followed by Bonferroni's post hoc test). (F, G) Levels of IL‐6 (F) and IL‐8 (G) in the culture medium of untreated or MLi‐2‐treated astrocytes derived from LRRK2‐PD iPSC lines, measured by ELISA. Data are presented as mean values ± SEM. *n* = 3, *n* = number of independent cell culture preparations; individual points represent the average of data from each single experiment (**p < 0.01, *p < 0.05 vs. LRRK2‐PD‐1; °*p* < 0.05 vs. LRRK2‐PD‐2; one‐way ANOVA followed by Bonferroni's post hoc test). (H) Quantitative PCR analysis of genes encoding BDNF, GDNF and NGF in untreated or MLi‐2‐treated astrocytes derived from LRRK2‐PD iPSC lines. Gene expression relative to GAPDH was assessed using the ΔCT method (RQ value) and was normalized to CTRL, which was arbitrarily set to a value of 100. Data are presented as mean values ± SEM. *n* = 3, *n* = number of independent cell culture preparations; individual points represent the average of data from each single experiment (***p < 0.001, *p < 0.05 vs. LRRK2‐PD‐1; °°°*p* < 0.001 vs. LRRK2‐PD‐2; one‐way ANOVA followed by Bonferroni's post hoc test). (I, J) Levels of BDNF (I) and NGF (J) released in the culture medium of iPSC‐derived astrocytes from untreated and MLi‐2‐treated astrocytes derived from LRRK2‐PD iPSC lines, measured by ELISA. Data are presented as mean values ± SEM. *n* = 3, *n* = number of independent cell culture preparations; individual points represent the average of data from each single experiment (**p < 0.01, *p < 0.05 vs. LRRK2‐PD‐1; °*p* < 0.05 vs. LRRK2‐PD‐2; one‐way ANOVA followed by Bonferroni's post hoc test).

To investigate the role of G2019S mutation in inducing astrocytes' detrimental effects, we normalized LRRK2 kinase activity and investigated their ability in supporting mouse DA neuron tropism. It is well known, in fact, that G2019S mutation, increasing LRRK2 kinase activity, leads to excessive autophosphorylation of LRRK2 and then, phosphorylation of specific substrates associated with PD pathogenesis, such as Rab proteins (Price et al. 2018; Lewis 2022). On these bases, treatment with LRRK2 inhibitors has been demonstrated to rescue the main pathological features observed in LRRK2‐derived neuronal cultures (Bailey and Cookson 2024; Bono et al. 2021; Lewis 2022; Oun et al. 2022; Weykopf et al. 2019). Cultures derived from LRRK2‐PD‐1 and LRRK2‐PD‐2 iPSC (Day 70) were treated with two different LRRK2 inhibitors, the GSK2578215A (GSK; 200 nM) or the MLi‐2 (500 nM) for 10 days. The ability of both GSK and MLi‐2 in inhibiting LRRK2 activity was assessed by measuring the levels of pSer935 LRRK and pSer106 Rab1 by Western blot in untreated and treated cells, showing that both pSer935 LRRK2 levels, but not total LRRK2 levels, and pSer106 Rab12, but not total Rab12, were significantly reduced by GSK and MLi‐2 (Figure S3). GSK‐treated, MLi‐2‐treated and untreated LRRK2‐PD‐1 and LRRK‐PD‐2 astrocytes were co‐cultured with mouse DA neurons for 7 days, followed by morphological analyses of TH‐positive neurons. As shown in Figures 7A and 8A, co‐culturing mouse DA neurons with GSK‐treated or MLi‐2‐treated LRRK2‐PD‐1 and LRRK2‐PD‐2 astrocytes significantly enhanced both dendrite length (Figures 7B and 8B, respectively) and dendrite number (Figures 7C and 8C, respectively) of TH‐positive neurons as compared to those observed when mouse DA neurons were co‐cultured with untreated LRRK2‐PD‐1 and LRRK2‐PD‐2 astrocytes. To exclude direct neuronal effects, parallel experiments were performed by incubating mouse DA neurons for 7 days with the same concentration of both GSK and MLi‐2. As shown in Figure S4, in morphological analyses of treated‐DA neurons no significant changes, either in dendrite length or in numbers, were observed, suggesting that in co‐culture experiments, LRRK2 inhibitors increase neuron morphology acting through astrocytes.

Untreated, GSK‐treated or MLi‐2‐treated LRRK2‐PD‐1 and LRRK2‐PD‐2 cultures were also analyzed for GFAP by immunofluorescence, showing the ability of both GSK and MLi‐2 to significantly reduce GFAP intensity (Figures 7D and 8D, respectively) as well as astrocytic territory size (Figures 7E and 8E, respectively). Interestingly, levels of soluble IL‐6 and IL‐8 were significantly reduced in the culture media of both GSK‐treated and MLi‐2‐treated LRRK2‐PD‐1 and LRRK2‐PD‐2 cultures compared to untreated ones (Figures 7F,G and 8F,G, respectively). Moreover, the mRNA levels for BDNF, GDNF, and NGF, as measured by qPCR, were significantly higher in both GSK‐treated and MLi‐2‐treated LRRK2‐PD‐1 and LRRK2‐PD‐2 cultures, with levels like those detected in CTRL cultures, compared to untreated cultures (Figures 7H and 8H, respectively). Consistently, increased secretion of BDNF and NGF in GSK‐treated and MLi‐2‐treated LRRK2‐PD‐1 and LRRK2‐PD‐2 culture media was measured by ELISA (Figures 7I,J and 8I,J, respectively).

**FIGURE 9:**
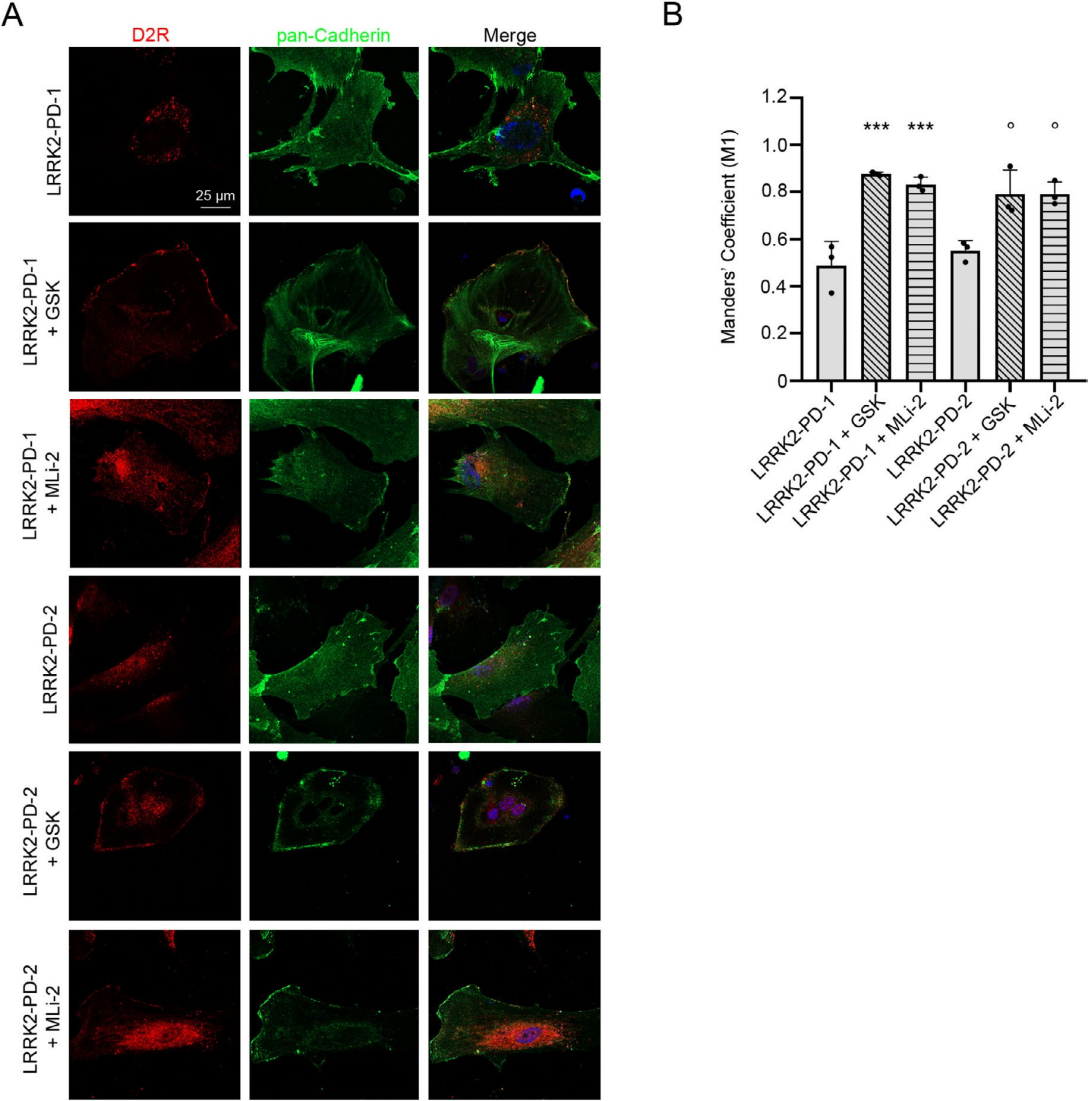
Effect of LRRK2 kinase activity inhibition on LRRK2‐PD‐derived astrocytes D2R localization. (A) Representative immunofluorescence images showing D2R (red) and pan‐cadherin (green) colocalization in iPSC‐derived astrocytes from untreated and GSK‐ or Mli‐2‐treated LRRK2‐PD lines. Nuclei were stained with DAPI (blue). (B) Quantification of D2R and cadherin colocalization in iPSC‐derived astrocytes from untreated and GSK‐ or Mli‐2‐treated LRRK2‐PD lines (day 70) performed using the Manders' overlap coefficient. Values range from 0 (no colocalization) to 1 (complete colocalization). Data are presented as mean values ± SEM. *n* = 3, *n* = number of independent cell culture preparations; individual points represent the average of data from each single experiment (***p < 0.001, *p < 0.05 vs. LRRK2‐PD‐1; °°°*p* < 0.001, °*p* < 0.05 vs. LRRK2‐PD‐2; one‐way ANOVA followed by Bonferroni's post hoc test).

Finally, immunofluorescence experiments showed that in LRRK2‐PD‐1 and LRRK‐PD‐2 astrocytes, treatment with both GSK and MLi‐2 restored the membrane localization of D2R. As shown in Figure 9, while the majority of untreated LRRK2‐PD‐1 and LRRK2‐PD‐2 cells displayed a reduced D2R‐ cadherins colocalization signal, both GSK and MLi‐2 treatment significantly restored the D2R localization at the plasma membrane (M1; LRRK2‐PD‐1 + GSK: +50% ± 0.6% of increase vs. LRRK2‐PD‐1; LRRK2‐PD‐1 + MLi‐2: +43.6% ± 3.0% of increase vs. LRRK2‐PD‐1; LRRK2‐PD‐2 + GSK: +23% ± 5.9% of increase vs. LRRK2‐PD‐2; LRRK2‐PD‐2 + MLi‐2: +31% ± 2.9% of increase vs. LRRK2‐PD‐1). Together, these data suggest that reducing the hyperactivation of G2019S LRRK2 reverses the pro‐inflammatory properties of LRRK2‐PD astrocytes toward a neurotrophic phenotype. Moreover, reducing the abnormal activation of LRRK2 is associated with the rescue of D2R cell membrane expression, allowing DA to promote D2R‐induced neuronal remodeling and decrease D2R‐induced neuroinflammation.

## 4 Discussion

In PD, evidence of astrocytes' reduced capability to sustain neuronal trophism, along with increased release of pro‐inflammatory cytokines and oxidative stress mediators has been provided, which likely contributes to the worsening of DA neuron damage (Harackiewicz and Grembecka 2024). To date, much of the understanding of astrocyte involvement in PD has been derived from studies conducted in animal models, mainly rodents, and more recently, from human astrocytes derived from iPSC with the advantage to overcome species‐specific differences, in terms of morphology, cellular complexity, and metabolic profile (Li et al. 2021; Pons‐Espinal et al. 2021; Zhang et al. 2016).

In this study, we generated an almost pure population of human astrocytes from an iPSC line derived from a healthy control. The differentiation process was validated by monitoring the expression of stage‐specific markers using both qPCR and immunofluorescence. By Day 70, cultures contained approximately 80% GFAP‐positive astrocytes that exhibited spontaneous intracellular calcium oscillations, indicative of their functional maturation (Bazargani and Attwell 2016; Evans et al. 2025; Khakh and McCarthy 2015; Nedergaard 1994; Parpura et al. 1994). Moreover, iPSC‐derived astrocytes were able to increase DA neuron arborization, as shown by co‐culture experiments with iPSC‐derived human astrocytes used to support mouse mesencephalic neurons. Similar results were obtained when DA neurons were incubated with astrocyte‐derived conditioned media, thus pointing to the involvement of secreted factors in sustaining DA neuron trophism. On this line, we found that iPSC‐derived astrocytes expressed high levels of mRNAs encoding growth factors, mainly BDNF and NGF, supporting their role in promoting neuronal survival, resilience, and regeneration under pathological conditions (Bailey and Cookson 2024; Miyazaki and Asanuma 2020). Intriguingly, accumulating evidence indicates that DA is involved in astrocytic release of growth factors (Miyazaki and Asanuma 2020; Broome Thomas et al. 2020) through the activation of their specific receptors, widely expressed on astrocytes in a region‐specific manner (Favetta and Bubacco 2025). Both D1 receptors (D1R) and D2 receptors (D2R) have been implicated in DA‐induced synthesis and release of growth factors (Miyazaki and Asanuma 2020; Broome Thomas et al. 2020), being the D2R stimulation particularly associated with NGF, BDNF, and GDNF production (Kinor et al. 2001; Kuric et al. 2013; Ohta et al. 2003, 2010) and, in turn, with DA neuron protection (Du et al. 2005; Hong et al. 2008; Imamura et al. 2008; Reuss and Unsicker 2000). Therefore, in cultures of human iPSC‐derived astrocytes, DAR expression and localization were analyzed showing that, in contrast with rodent models (Miyazaki et al. 2004), the mRNAs encoding only D1R and D2R, but not D3R, D4R and D5R were expressed. Moreover, immunofluorescence experiments showed that in human astrocytes D1R and D2R were highly co‐localized with cadherin, used as a plasma membrane marker, pointing to their greatly localization at this cell site.

To dissect the contribution of astrocyte D1R and D2R in inducing neurotrophic effects, co‐culture experiments combined with the use of the selective D1R and D2R antagonists, SCH23990 and sulpiride, were developed, showing the crucial role and specific role of D2R in increasing DA neurons' arborization. In line with previous data, these findings highlight a key role for astrocytic D2R in promoting neuronal support (Miyazaki and Asanuma 2020) implying that L‐DOPA and D2R agonists, used in PD therapy, may exert neuroprotective effects at least in part through astrocytic D2R activation and neurotrophic factor production (Broome Thomas et al. 2020; Gelders et al. 2018; Kinor et al. 2001; Kuric et al. 2013; Ohta et al. 2000, 2003, 2010).

Chronic neuroinflammation is known to be involved in the pathophysiology of PD (Miyazaki and Asanuma 2020; Possemato et al. 2023; Prunell and Olivera‐Bravo 2022) and the LRRK2 gene is crucial for their function (Booth et al. 2017). Interestingly, mutations in this gene have been particularly associated with an enhanced inflammatory response by astrocytes, as reported in both patients and PD animal models (Bailey and Cookson 2024; Ho et al. 2024; Pons‐Espinal et al. 2024). Recently, we obtained DA neurons from iPSC lines derived from two PD patients carrying G2019S LRRK2 mutation (LRRK2‐PD iPSC lines), together with their isogenic counterparts in which the mutation has been corrected (Bono et al. 2021). As reported, the analysis of iPSC‐derived cultures has shown that a main characteristic of DA neurons with G2019S LRRK2 mutation was a significant reduction of D3R and nAChR membrane localization due to impairment in their trafficking from intracellular sites to the plasma membrane (Bono et al. 2021). In this study LRRK2‐PD‐1 and LRRK2‐PD‐2 iPSC lines, their respective gene‐corrected iPSC, and a control iPSC line were used to obtain cultures of human astrocytes to be fully characterized. Each LRRK2‐PD iPSC line was independently examined, with superimposable results. We found that G2019S mutation in LRRK2 did not impair the differentiation of iPSC into astrocytes, as assessed by the percentage of GFAP‐positive cells and the spontaneous calcium activity observed at the end of the differentiation protocol (Day 70). However, compared to astrocytes derived from isogenic corrected iPSC lines and from healthy controls, those carrying the LRRK2 mutation were hypertrophic and displayed increased levels of GFAP at both mRNA and protein levels, two canonical hallmarks of reactive astrocytes (Escartin et al. 2021). Moreover, iPSC‐derived LRRK2‐PD astrocytes, but not isogenic or control ones, exhibited a pro‐inflammatory phenotype, as evidenced by increased secretion of IL‐6 and IL‐8. On this line, high levels of pro‐inflammatory cytokines such as TNF, IL‐1β, IL‐2, IL‐4, and IL‐6 have been reported in the culture medium of astrocytes carrying the G2019S LRRK2 mutation (Ho et al. 2024; Pons‐Espinal et al. 2024). Notably, IL‐6 levels have been specifically associated with dopaminergic neurodegeneration (Pons‐Espinal et al. 2024). Moreover, increased levels of those cytokines have been detected in post‐mortem brain tissues from PD patients as well as in patients' serum (Allan and Pinteaux 2003; Hofmann et al. 2009; Hunot et al. 1999; Mogi et al. 1994, 1996; Pons‐Espinal et al. 2024). Interestingly, peripheral inflammation has been found in serum samples of asymptomatic G2019S mutation carriers, genetically at risk of developing the disease (Dzamko et al. 2016), suggesting that in these individuals, an abnormal astrocyte activity may represent a very early pathogenetic event that potentially triggers the neurodegeneration process. Moreover, these data also suggest that in G2019S LRRK2 PD patients the neuroinflammatory component may be even greater than in other PD patients (Brockmann et al. 2017; Dzamko et al. 2016). By contrast, the ability to synthesize neurotrophic factors such as BDNF, GDNF, and NGF, observed in control iPSC‐derived astrocytes and in astrocytes derived from gene‐corrected iPSC, was completely lost in LRRK2‐PD astrocytes. This is in line with other observations showing an association between G2019S mutation in the LRRK2 gene and the inability of astrocytes to release in growth factors, such as NGF (Ho et al. 2024), possibly due to an impairment in vesicle biogenesis and trafficking, including those containing trophic factors (de Rus Jacquet et al. 2021). Intriguingly, several evidence have shown that astrocytic D2R, besides their role in controlling the release of growth factors, also suppress neuroinflammation (Broome Thomas et al. 2020; Favetta and Bubacco 2025; Possemato et al. 2023). For example, D2R stimulation by agonists resulted in a marked reduction in the levels of astrocyte activation, including IL‐6, thus preventing DA neuron loss upon neurotoxic exposure (Morimoto et al. 2022; Shao et al. 2013). Moreover, D2R activation has been shown to inhibit the NOD‐like receptor family pyrin domain‐containing 3 (NLRP3) inflammasome pathway and the subsequent release of pro‐inflammatory cytokines such as the IL‐1β production, strongly implicated in DA neuron death (Zhu et al. 2018). On this line, it is well known that D2R, like many other GPCRs, signals through both G proteins and β‐arrestins is now well accepted (Beaulieu and Gainetdinov 2011). In astrocytes, the mechanism that may underlie D2R‐dependent release of growth factors likely involves the G‐protein‐beta/gamma subunits that, activating the phospholipase C, lead to an increase in inositol trisphosphate and thus in intracellular calcium (Agulhon et al. 2008; Ahmadiantehrani and Ron 2013). By contrast, D2R‐induced inhibition of neuroinflammation likely involves a beta arrestin2‐dependent mechanism (Zhu et al. 2018), that is significantly affected by diffuse α‐syn, implying that besides PD, other synucleinopathies may be characterized by high levels of neuroinflammation due to the loss of D2R‐mediated anti‐inflammation properties (Du et al. 2018). The expression level and localization of DAR were thus analyzed, especially focusing on D2R, showing comparable D2R mRNA levels in astrocytes derived from all iPSC lines. However, in most astrocytes carrying G2019S LRRK2 mutation, D2R colocalization with cadherins in immunofluorescence and confocal analyses, and cell‐surface biotinylation assays clearly showed a reduced D2R plasma membrane localization suggesting that, as shown in neurons (Bono et al. 2021), an altered trafficking to the plasma membrane is a characteristic of astrocytes with this specific mutation. On this line, the role of LRRK2 in regulating protein trafficking has been clearly established (Bailey and Cookson 2024) and mutation in LRRK2 has been shown to impact DA receptor trafficking in both transfected cell lines and in primary neurons derived from transgenic mouse models (Migheli et al. 2013; Rassu et al. 2017; Tong et al. 2009). Interestingly, in primary mouse astrocytes, G2019S LRRK2 mutation impairs the functionality of EAAT2 by disrupting the Rab‐dependent recycling to the plasma membrane and promoting intracellular retention and degradation (Iovino et al. 2022). Although not yet investigated, a mutation‐induced impairment of the endolysosomal pathway may likewise contribute to the reduced plasma‐membrane localization of astrocytic D2R.

From a functional point of view, as compared to iPSC‐derived astrocytes with the gene correction or control astrocytes, those carrying the LRRK2 mutation, or their conditioned medium, totally lost their capability to support DA neurons trophism: mouse DA neurons co‐cultured with astrocytes or incubated with conditioned media, in fact, were characterized by a marked reduction in dendritic arborization. This effect is attributable to a reduced availability of growth factors and/or to an increased release of inflammatory cytokines associated with the LRRK2 mutation, along with the inability of DA to act as neuromodulator factor through the D2R‐mediated trophic and anti‐inflammatory effects. Since G2019S mutation enhances LRRK2 kinase activity by increasing the enzyme's catalytic rate (Thomas and Beal 2007), that, in turn, contributes to cellular toxicity (Greggio et al. 2009), LRRK2 inhibitors has been widely employed as a pharmacological strategy to rescue structural and functional deficits in various PD models (Weykopf et al. 2019; Bono et al. 2021; Pons‐Espinal et al. 2024). In this study, the GSK and the MLi‐2 inhibitors were used to reduce G2019S LRRK2 kinase hyperactivity in iPSC‐derived LRRK2‐PD astrocytes, followed by molecular, cellular and functional analyses. We found that LRRK2 inhibitor‐treated LRRK2‐PD astrocytes lost their reactive phenotype, as evidenced by reduced GFAP expression, decreased hypertrophy, and diminished secretion of pro‐inflammatory cytokines IL‐6 and IL‐8. By contrast, treated LRRK2‐PD astrocytes gained a protective phenotype, characterized by the release of neurotrophic factors such as BDNF and NGF, and the ability to promote DA neuron remodeling. Intriguingly, LRRK2 inhibition also restored D2R localization at the plasma membrane, thus restoring both the D2R‐mediated release of growth factors and the D2R‐mediated suppression of proinflammatory cytokines.

## 5 Conclusion

**FIGURE 10:**
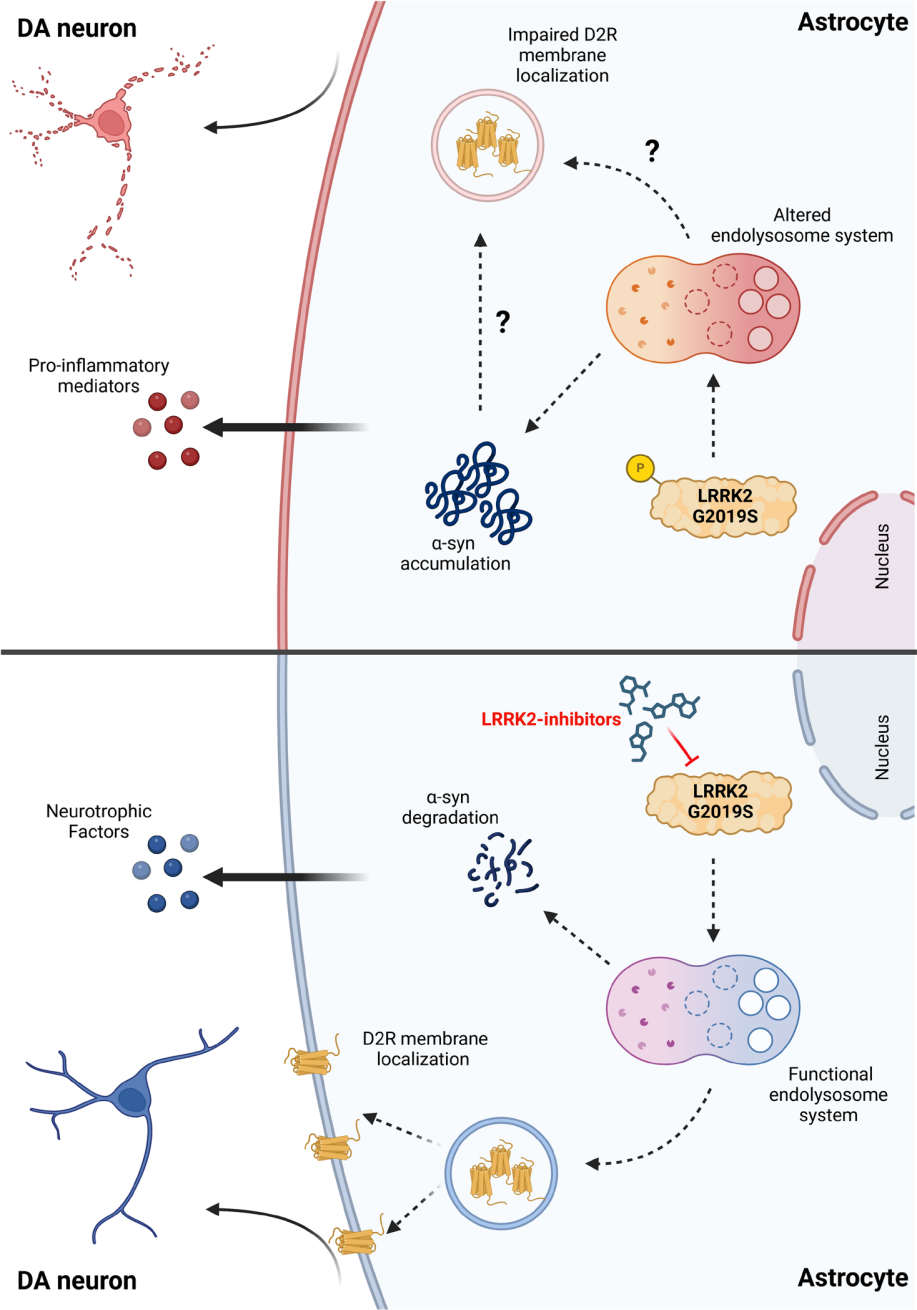
G2019S LRRK2 impairs D2R membrane localization in astrocytes. Schematic representation of the proposed mechanism underlying intracellular retention of D2R (created with BioRender.com).

DA represents a key modulator of astrocyte function, as activation of DA receptors can influence both neurotrophic and anti‐inflammatory responses. In this study, using a human model of iPSC‐derived astrocytes, we showed that these cells express both D1R and D2R, with the latter being mainly involved in supporting DA neuron trophism. Interestingly, we also found that D2R trafficking to the astrocyte membrane is significantly affected by the G2019S mutation in LRRK2, a common variant implicated in both genetic and sporadic PD pathogenesis. Thus, on one hand, this mutation profoundly alters the phenotype of astrocytes that shifts from a neurotrophic to a pro‐inflammatory profile (Di Domenico et al. 2019; Ho et al. 2025). On the other hand, dysregulation of LRRK2 activity may directly impair D2R trafficking, thus compromising the protective DA signaling and leading to exacerbation of the neuroinflammation processes and neuronal damage; moreover, abnormal α‐syn has been shown to impair D2R function, thus representing an additional mechanism that leads to reduced astrocytic D2R signaling (Du et al. 2018; Shao et al. 2013) (Figure 10). Therefore, astrocytic D2R dysfunction may be a common pathogenetic trait of PD and, more in general, of synucleinopathies. Future experiments will examine D2R localization in astrocytes derived from iPSC lines harboring mutations in additional PD–associated genes and available in our laboratory. This is also relevant from a therapeutic point of view: in PD patients, therapies with L‐dopa and D2R agonists could be useful to keep neuroinflammation under control, unless in those PD patients with reduced astrocyte D2R activity, such as those carrying the G2019S mutation in LRRK2. In these PD patients, LRRK2 kinase inhibitors, as well as other pharmacological approaches aimed at controlling LRRK2 hyperactivity, are currently under clinical investigation as pathway‐specific and personalized therapeutic strategies (Bono et al. 2022; Leiter et al. 2026).

Intriguingly, similar alterations in astrocytic D2R have been observed in the hippocampus and striatum area of animal models of Huntington's disease as well as in patient post‐mortem brain tissues (Harris et al. 2022), thus suggesting that astrocytic D2R dysregulation may represent a shared pathological feature across multiple neurodegenerative and neuropsychiatric disorders that need to be treated (Verkhratsky et al. 2023).

## Author Contributions

**Chiara Gnutti:** investigation, formal analysis. **Veronica Mutti:** investigation, methodology, formal analysis, writing – original draft. **Silvia Bolognin:** resources, writing – review and editing. **Daniela Bosisio:** writing – review and editing. **Giulia Sbrini:** investigation, formal analysis. **Zaira Tomasoni:** investigation, formal analysis. **Isabella Russo:** writing – review and editing. **Federica Bono:** investigation, writing – original draft, methodology, formal analysis, funding acquisition. **Chiara Fiorentini:** conceptualization, writing – review and editing, formal analysis, supervision, funding acquisition. **Valentina Salvi:** investigation, formal analysis. **Cristina Missale:** writing – review and editing. **Carolina Gaudenzi:** investigation, formal analysis. **Dounia Fadel:** investigation, formal analysis. **Jens C. Schwamborn:** resources, writing – review and editing.

## Funding

This work was supported by grants from the University of Brescia, Italy to F.B., C.M., and C.F.

## Conflicts of Interest

The authors declare no conflicts of interest.

## Supporting information


**Figure S1:** (A) Representative image of blots of LRRK2 phosphorylation at Ser935 (pS935 LRRK2), total LRRK2, Rab12 phosphorylation at Ser106 (pS106 Rab12), total Rab12 and GAPDH in iPSC‐derived astrocytes from CTRL, LRRK2‐PD, and LRRK2‐ISO lines. (B) Densitometric analysis of western blots with specific levels of pS935 LRRK2 normalized to the corresponding total LRRK2 and GAPDH levels. (C) Densitometric analysis of Western blots with specific levels of pS106 Rab12 normalized to the corresponding total Rab12 and GAPDH levels. Data are presented as mean values ± SEM. *n* = 3, *n* = number of independent cell culture preparations; individual points represent the average of data from each single experiment (***p < 0.001, **p < 0.01, *p < 0.05 vs. CTRL; one‐way ANOVA followed by Bonferroni's post hoc test).
**Figure S2:** Functional characterization and gene expression analysis of iPSC‐derived astrocytes obtained from two PD patients carrying the LRRK2 G2019S mutation. (A, B) Functional characterization of iPSC‐derived astrocytes using the green fluorescent cell‐permeable calcium indicator, Fluo‐4 AM. Spontaneous calcium wave recording for 300 s (s) (*n* = 3) in astrocytes derived from LRRK2‐PD‐1 and LRRK2‐ISO‐1 (A), and from LRRK2‐PD‐2 and LRRK2‐ISO‐2 (B) iPSC lines. (C, D) Quantitative PCR analysis of S100β (C) and GLAST (D) mRNA expression in iPSC‐derived astrocytes from CTRL, LRRK2‐PD and LRRK2‐ISO lines. Gene expression relative to GAPDH was assessed using the ΔΔCT method (RQ value) and was normalized to CTRL, which was arbitrarily set to a value of 100. Data are presented as mean values ± SEM. *n* = 3, *n* = number of independent cell culture preparations; individual points represent the average of data from each single experiment.
**Figure S3:** (A) Representative image of blots of LRRK2 phosphorylation at Ser935 (pS935 LRRK2), total LRRK2, Rab12 phosphorylation at Ser106 (pS106 Rab12), total Rab12 and GAPDH in untreated and GSK‐ or MLi‐2‐treated iPSC‐derived astrocytes from LRRK2‐PD lines. (B) Densitometric analysis of western blots with specific levels of pS935 LRRK2 normalized to the corresponding total LRRK2 and GAPDH levels. (C) Densitometric analysis of western blots with specific levels of pS106 Rab12 normalized to the corresponding total Rab12 and GAPDH levels. Data are presented as mean values ± SEM. *n* = 3, *n* = number of independent cell culture preparations; individual points represent the average of data from each single experiment (***p < 0.001 vs. LRRK2‐PD‐1; °°°*p* < 0.001 vs. LRRK2‐PD‐2; one‐way ANOVA followed by Bonferroni's post hoc test).
**Figure S4:** (A, B) Morphological analysis of primary dendrite length (A) and dendrite number (B) of untreated and GSK‐ or MLi‐2‐treated mDA neurons. Data are presented as mean values ± SEM. *n* = 3, *n* = number of independent cell culture preparations; individual points represent the average of data from each single experiment.
**Table S1:** List of patients enrolled in this study.
**Table S2:** List of antibodies used in immunofluorescence (IF), immunocytochemistry (ICC), or western blot (WB) analysis.
**Table S3:** List of secondary antibodies used in immunofluorescence (IF) analysis.
**Table S4:** List of primers used in qPCR analysis.
**Table S5:** Statistical summary of data in Figure 2B,C. Statistical significance was determined by one‐way ANOVA followed by the Bonferroni's post hoc test. Values indicate *F* values (F), degrees of freedom (df) and *p* values. *p* < 0.05 was considered statistically significant.
**Table S6:** Statistical summary of data in Figure 3E,F. Statistical significance was determined by one‐way ANOVA followed by the Bonferroni's post hoc test. Values indicate *F* values (*F*), degrees of freedom (df) and *p* values. *p* < 0.05 was considered statistically significant.
**Table S7:** Statistical summary of data in Figure 4C–K. Statistical significance was determined by one‐way ANOVA followed by the Bonferroni's post hoc test. Values indicate *F* values (*F*), degrees of freedom (df) and *p* values. *p* < 0.05 was considered statistically significant.
**Table S8:** Statistical summary of data in Figure 5C. Statistical significance was determined by one‐way ANOVA followed by the Bonferroni's post hoc test. Values indicate *F* values (*F*), degrees of freedom (df) and *p* values. *p* < 0.05 was considered statistically significant.
**Table S9:** Statistical summary of data in Figure 6B,C,E,F. Statistical significance was determined by one‐way ANOVA followed by the Bonferroni's post hoc test. Values indicate *F* values (*F*), degrees of freedom (df) and *p* values. *p* < 0.05 was considered statistically significant.
**Table S10:** Statistical summary of data in Figure 7B–J. Statistical significance was determined by one‐way ANOVA followed by the Bonferroni's post hoc test. Values indicate *F* values (*F*), degrees of freedom (df) and *p* values. *p* < 0.05 was considered statistically significant.
**Table S11:** Statistical summary of data in Figure 8B–J. Statistical significance was determined by one‐way ANOVA followed by the Bonferroni's post hoc test. Values indicate *F* values (*F*), degrees of freedom (df) and *p* values. *p* < 0.05 was considered statistically significant.
**Table S12:** Statistical summary of data in Figure 9B. Statistical significance was determined by one‐way ANOVA followed by the Bonferroni's post hoc test. Values indicate *F* values (*F*), degrees of freedom (df) and *p* values. *p* < 0.05 was considered statistically significant.
**Table S13:** Statistical summary of data in Figure S1B,C. Statistical significance was determined by one‐way ANOVA followed by the Bonferroni's post hoc test. Values indicate *F* values (*F*), degrees of freedom (df) and *p* values. *p* < 0.05 was considered statistically significant.
**Table S14:** Statistical summary of data in Figure S3B,C. Statistical significance was determined by one‐way ANOVA followed by the Bonferroni's post hoc test. Values indicate *F* values (*F*), degrees of freedom (df) and *p* values. *p* < 0.05 was considered statistically significant.

## Data Availability

The data that support the findings of this study are available from the corresponding author upon reasonable request.
