# Supplementary material for: LRRK2 Mutation Alters Dopamine D2 Receptor Localization in Induced Pluripotent Stem Cells‐Derived Astrocytes From Parkinson's Disease Patients: Implications for Neuronal Damage
Source: J Neurochem. 2026 Jul 1;170(7):e70510. doi: 10.1111/jnc.70510 (PMC13320301; doi:10.1111/jnc.70510)
Supplement: Supplementary file 1 — Figure S1: (A) Representative image of blots of LRRK2 phosphorylation at Ser935 (pS935 LRRK2), total LRRK2, Rab12 phosphorylation at Ser106 (pS106 Rab12), total Rab12 and GAPDH in iPSC‐derived astrocytes from CTRL, LRRK2‐PD, and LRRK2‐ISO lines. (B) Densitometric analysis of western blots with specific levels of pS935 LRRK2 normalized to the corresponding total LRRK2 and GAPDH levels. (C) Densitometric analysis of Western blots with specific levels of pS106 Rab12 normalized to the corresponding total Rab12 and GAPDH levels. Data are presented as mean values ± SEM. n = 3, n = number of independent cell culture preparations; individual points represent the average of data from each single experiment (***p < 0.001, **p < 0.01, *p < 0.05 vs. CTRL; one‐way ANOVA followed by Bonferroni's post hoc test). Figure S2: Functional characterization and gene expression analysis of iPSC‐derived astrocytes obtained from two PD patients carrying the LRRK2 G2019S mutation. (A, B) Functional characterization of iPSC‐derived astrocytes using the green fluorescent cell‐permeable calcium indicator, Fluo‐4 AM. Spontaneous calcium wave recording for 300 s (s) (n = 3) in astrocytes derived from LRRK2‐PD‐1 and LRRK2‐ISO‐1 (A), and from LRRK2‐PD‐2 and LRRK2‐ISO‐2 (B) iPSC lines. (C, D) Quantitative PCR analysis of S100β (C) and GLAST (D) mRNA expression in iPSC‐derived astrocytes from CTRL, LRRK2‐PD and LRRK2‐ISO lines. Gene expression relative to GAPDH was assessed using the ΔΔCT method (RQ value) and was normalized to CTRL, which was arbitrarily set to a value of 100. Data are presented as mean values ± SEM. n = 3, n = number of independent cell culture preparations; individual points represent the average of data from each single experiment. Figure S3: (A) Representative image of blots of LRRK2 phosphorylation at Ser935 (pS935 LRRK2), total LRRK2, Rab12 phosphorylation at Ser106 (pS106 Rab12), total Rab12 and GAPDH in untreated and GSK‐ or MLi‐2‐treated iPSC‐derived astrocytes from LRRK2‐P [file JNC-170-e70510-s001.pdf]

Supporting information

LRRK2 mutation alters dopamine D2 receptor localization in induced pluripotent stem cells-derived astrocytes from Parkinson's disease patients: implications for neuronal damage

Veronica Mutti[1]*, Federica Bono[1]*#, Zaira Tomasoni[1], Dounia Fadel[1], Chiara Gnutti[1], Giulia Sbrini[1], Carolina Gaudenzi[1], Valentina Salvi[1], Daniela Bosisio[1], Isabella Russo[1], Silvia Bolognin[2], Jens C. Schwamborn[3], Cristina Missale[1], Chiara Fiorentini[1].

[1]Department of Molecular and Translational Medicine, University of Brescia, Brescia, Italy.
[2]MERLN Institute for Technology-Inspired Regenerative Medicine, Maastricht University, Maastricht, The Netherlands.
[3]Developmental and Cellular Biology, Luxembourg Centre for Systems Biomedicine, University of Luxembourg, Belvaux, Luxembourg

* First co-authors
# Corresponding author

Supplementary Figures

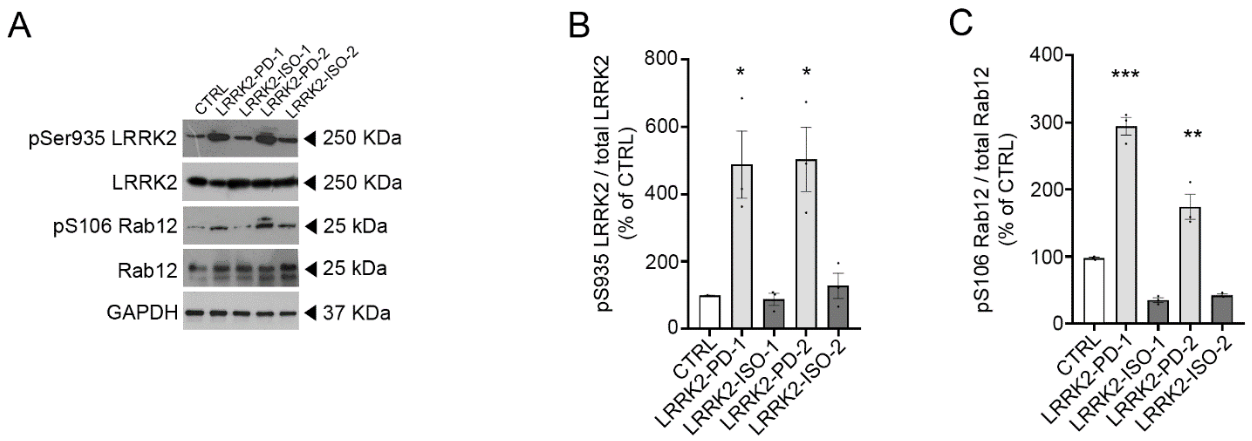

Fig. S1:

(A) Representative image of blots of LRRK2 phosphorylation at Ser935 (pS935 LRRK2), total LRRK2, Rab12 phosphorylation at Ser106 (pS106 Rab12), total Rab12 and GAPDH in iPSC-derived astrocytes from CTRL, LRRK2-PD and LRRK2-ISO lines. (B) Densitometric analysis of Western blots with specific levels of pS935 LRRK2 normalized to the corresponding total LRRK2 and GAPDH levels. (C) Densitometric analysis of Western blots with specific levels of pS106 Rab12 normalized to the corresponding total Rab12 and GAPDH levels. Data are presented as mean values ± SEM. n = 3., n = number of independent cell culture preparations; individual points represent the average of data from each single experiment (***p<0.001, **p<0.01, *p<0.05 vs CTRL; one-way ANOVA followed by Bonferroni's post hoc test).

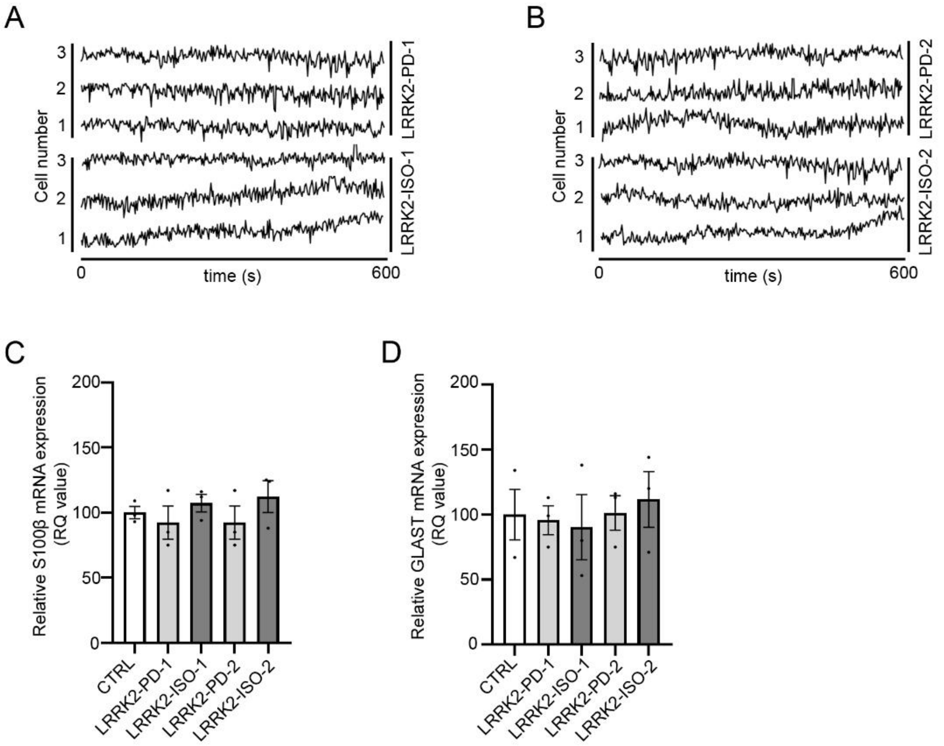

Fig. S2: Functional characterization and gene expression analysis of iPSC-derived astrocytes obtained from two PD patients carrying the LRRK2 G2019S mutation. (A-B) Functional characterization of iPSC-derived astrocytes using the green fluorescent cell-permeable calcium indicator, Fluo-4 AM. Spontaneous calcium wave recording for 300 seconds (s) (n=3) in astrocytes derived from LRRK2-PD-1 and LRRK2-ISO-1 (A), and from LRRK2-PD-2 and LRRK2-ISO-2 (B) iPSC lines. (C-D) Quantitative PCR analysis of S100β (C) and GLAST (D) mRNA expression in iPSC-derived astrocytes from CTRL, LRRK2-PD and LRRK2-ISO lines. Gene expression relative to GAPDH was assessed using the ΔΔCT method (RQ value) and was normalized to CTRL, which was arbitrarily set to a value of 100. Data are presented as mean values ± SEM. n = 3., n = number of independent cell culture preparations; individual points represent the average of data from each single experiment.

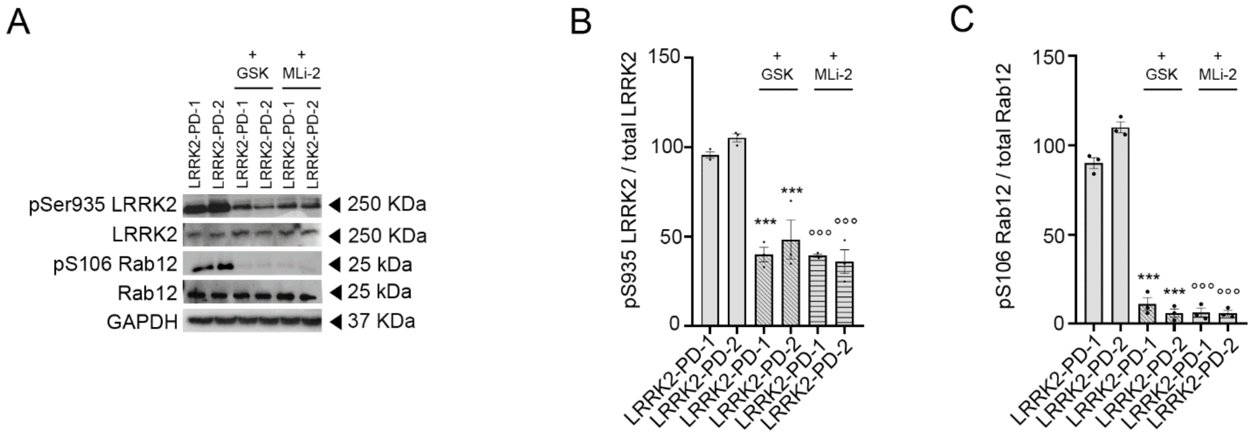

Fig. S3:

(A) Representative image of blots of LRRK2 phosphorylation at Ser935 (pS935 LRRK2), total LRRK2, Rab12 phosphorylation at Ser106 (pS106 Rab12), total Rab12 and GAPDH in untreated and GSK- or MLi-2-treated iPSC-derived astrocytes from LRRK2-PD lines. (B) Densitometric analysis of Western blots with specific levels of pS935 LRRK2 normalized to the corresponding total LRRK2 and GAPDH levels. (C) Densitometric analysis of Western blots with specific levels of pS106 Rab12 normalized to the corresponding total Rab12 and GAPDH levels. Data are presented as mean values ± SEM. n = 3., n = number of independent cell culture preparations; individual points represent the average of data from each single experiment (***$p<0.001$ vs LRRK2-PD-1; °°°$p<0.001$ vs LRRK2-PD-2; one-way ANOVA followed by Bonferroni’s post hoc test).

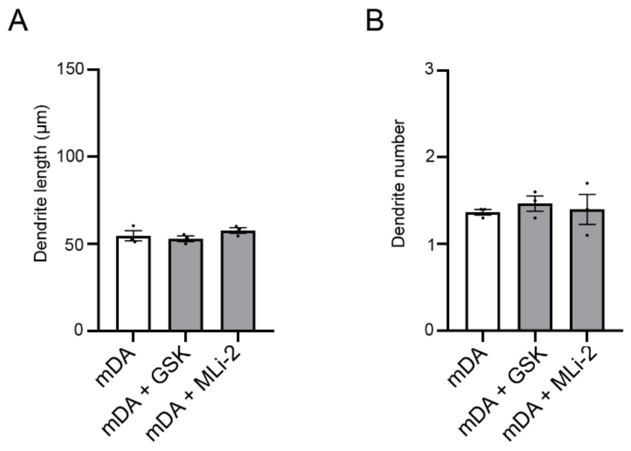

Fig. S4

(A-B) Morphological analysis of primary dendrite length (A) and dendrite number (B) of untreated and GSK- or MLi-2-treated mDA neurons. Data are presented as mean values ± SEM. n = 3., n = number of independent cell culture preparations; individual points represent the average of data from each single experiment.

Supplementary Tables

LINE ID	ORIGINAL LINE NAME	KNOWN MUTATION	PD STATUS	AGE	SEX	SOURCE & REFERENCE
CTRL	C2	None	Healthy	81	F	Reinhardt e al., 2013
LRRK2-PD-1	L1-2Mut	G2019S LRRK2	Parkinson's disease	54	F	Reinhardt e al., 2013
LRRK2-ISO-1	L1-2GC	None	Gene edited control	54	F	Reinhardt e al., 2013
LRRK2-PD-2	L2-3Mut	G2019S LRRK2	Parkinson's disease	81	F	Reinhardt e al., 2013
LRRK2-ISO-2	L2-3GC	None	Gene edited control	81	F	Reinhardt e al., 2013

Table S1: List of patients enrolled in this study

ANTIBODY	SOURCE	COMPANY	Cat. N°	APPLICATION	WORKING DILUITION
D1R	Rabbit	Invitrogen	PA5-27172	IF	1:100
D2R	Rabbit	Santa Cruz	sc-9113	IF/WB	1:100
GAPDH	Mouse	Invitrogen	MA5-157738	WB	1:10000
GFAP	Rabbit	Dako	Z0334	IF	1:1000
GFAP	Rabbit	GeneTex	GTX108711	WB	1:5000
GLAST	Rabbit	Novusbio	NB100-1869	IF	1:500
LRRK2	Rabbit	Abcam	ab133474	WB	1:10000
Na+K+ ATPase	Mouse	Thermo Fisher	MA3-915	WB	1:1000
Nestin	Rabbit	Millipore	ABD69	IF	1:400
pan-Cadherin	Mouse	Abcam	ab6528	IF	1:200
pSer106 Rab12	Rabbit	Abcam	ab256487	WB	1:1000
pSer935 LRRK2	Rabbit	Abcam	ab133450	WB	1:1000
Rab12	Rabbit	Proteintech	18843-1-AP	WB	1:1000
S100β	Rabbit	Abcam	ab227914	IF	1:100
SOX2	Rat	Thermo Fisher	740013T	IF	1:100
TH	Rabbit	Millipore	AB152	ICC	1:700

Table S2: List of antibodies used in immunofluorescence (IF), immunocytochemistry (ICC) or western blot (WB) analysis.

ANTIBODY	SOURCE	COMPANY	Cat. N°	WORKING DILUITION
Anti-mouse 488	Goat	Jackson Immuno Research	115-545-003	1:1000
Anti-rabbit 488	Goat	Jackson Immuno Research	111-545-003	1:1000
Anti-rat 488	Goat	Jackson Immuno Research	112-545-003	1:1000
Anti-mouse 594	Goat	Jackson Immuno Research	115-585-003	1:1000
Anti-rabbit 594	Goat	Jackson Immuno Research	111-585-003	1:1000
Anti-rat 594	Goat	Jackson Immuno Research	112-585-003	1:1000

Table S3: List of secondary antibodies used in immunofluorescence (IF) analysis.

**TARGET**	**FORWARD/REVERSE PRIMER (5′-3′)**
BDNF	TTCCACCAGGTGAGAAGAGT/<br>ACTAATACTGTCACACACGC
D1R	CCCGTTGTTGTTGATCTTA/<br>CTGTATGGTGCCCTTCTGTG
D2R	GAAAGCCACTCAGATGCTCG/<br>GCAGTGGAGGATCTTCAGGA
D3R	GTCTGGGTACTGGCCTTTGC/<br>AGTCACTCCAAAGGGCAGGT
D4R	CCTTCTTCGTGGTGCACAT/<br>AACTCGGCGTTGAAGACAGT
D5R	TCTCCTTCATTCCGGTCCAG/<br>AGGTCACGATCATGATGGCA
GAPDH	AGGTCGGAGTCAACGGATTT/<br>ATCTCGCTCCTGGAAGATGG
GDNF	TGAGCAGTGACTCAAATATG/<br>GTAAACCAGGTTATCATCTA
GFAP	GTCCCCCACCTAGTTTGCAG/<br>TAGTCGTTGGCTTCGTGCTT
GLAST	GGAAGGGCACAAAGGAAA/<br>CCCCCCAATCACACCCAT
MAP2	CCCCTTGCTTCCCTGTAGAA/<br>ATTTCCTCCTGGCAACCTCA
Nestin	TCTTTGCTCCCAGTCCTGAG/<br>GGGCTCTGATCTCTGCATCT
NGF	CACACTGAGGTGCATAGCGT/<br>TGATGACCGCTTGCTCCTGT
S100β	TGTAGACCCTAACCCGGAGG/<br>TGCATGGATGAGGAACGCAT
SOX2	GGGAAATGGGAGGGGTGCAAAAGAGG/<br>TTGCGTGAGTGTGGATGGGATTGGTG
TH	CCGAGCTGTGAAGGTGTTTG/<br>ACACTTGTCCAGCTCTGACA

Table S4: List of primers used in qPCR analysis.

Statistical summary

Figure	Analysis	Comparison	F(df)	Adjusted p-value
2B	One-way ANOVA	Overall effect	17.91 (2,6)	0.003
	Bonferroni post-hoc	mDA vs mDA+Astrocytes	-	0.0031
	Bonferroni post-hoc	mDA vs mDA+Medium	-	0.0274
2C	One-way ANOVA	Overall effect	10.76 (2,6)	0.0104
	Bonferroni post-hoc	mDA vs mDA+Astrocytes	-	0.0179
	Bonferroni post-hoc	mDA vs mDA+Medium	-	0.0250

Table S5: Statistical summary of data in Figure 2B-C. Statistical significance was determined by one-way ANOVA followed by the Bonferroni's post-hoc test. Values indicate F values (F), degrees of freedom (df) and p values. $p < 0.05$ was considered statistically significant.

Figure	Analysis	Comparison	F(df)	Adjusted p-value
3E	One-way ANOVA	Overall effect	14.38 (5,13)	<0.0001
	Bonferroni post-hoc	mDA vs mDA+Astrocytes	-	0.0143
	Bonferroni post-hoc	mDA vs mDA+Astrocytes+SCH	-	0.0005
3F	One-way ANOVA	Overall effect	17.49 (5,13)	<0.0001
	Bonferroni post-hoc	mDA vs mDA+Astrocytes	-	0.0003
	Bonferroni post-hoc	mDA vs mDA+Astrocytes+SCH	-	0.0037

Table S6: Statistical summary of data in Figure 3E-F. Statistical significance was determined by one-way ANOVA followed by the Bonferroni's post-hoc test. Values indicate F values (F), degrees of freedom (df) and p values. $p < 0.05$ was considered statistically significant.

Figure	Analysis	Comparison	F(df)	Adjusted p-value
4C	One-way ANOVA	Overall effect	5.13 (4,10)	0.02
	Bonferroni post-hoc	CTRL vs LRRK2-PD-1	-	<0.0001
	Bonferroni post-hoc	CTRL vs LRRK2-PD-2	-	<0.0001
4D	One-way ANOVA	Overall effect	31.25 (4,10)	<0.0001
	Bonferroni post-hoc	CTRL vs LRRK2-PD-1	-	<0.0001
	Bonferroni post-hoc	CTRL vs LRRK2-PD-2	-	0.0004
4E	One-way ANOVA	Overall effect	14.98 (4,10)	0.0003
	Bonferroni post-hoc	CTRL vs LRRK2-PD-1	-	0.0064
	Bonferroni post-hoc	CTRL vs LRRK2-PD-2	-	0.0008
4F	One-way ANOVA	Overall effect	14.33 (4,10)	0.0004
	Bonferroni post-hoc	CTRL vs LRRK2-PD-1	-	0.0141
	Bonferroni post-hoc	CTRL vs LRRK2-PD-2	-	0.0053
4G	One-way ANOVA	Overall effect	9.239 (4,10)	0.0022
	Bonferroni post-hoc	CTRL vs LRRK2-PD-1	-	0.0122

	Bonferroni post-hoc	CTRL vs LRRK2-PD-2	-	0.0014
4H	One-way ANOVA	Overall effect	10.33 (4,10)	0.0001
	Bonferroni post-hoc	CTRL vs LRRK2-PD-1	-	0.0276
	Bonferroni post-hoc	CTRL vs LRRK2-PD-2	-	0.0009
4I BDNF	One-way ANOVA	Overall effect	62.22 (4,10)	<0.0001
	Bonferroni post-hoc	CTRL vs LRRK2-PD-1	-	<0.0001
	Bonferroni post-hoc	CTRL vs LRRK2-PD-2	-	<0.0001
4I GDNF	One-way ANOVA	Overall effect	170.6 (4,109	<0.0001
	Bonferroni post-hoc	CTRL vs LRRK2-PD-1	-	<0.0001
	Bonferroni post-hoc	CTRL vs LRRK2-PD-2	-	<0.0001
4I NGF	One-way ANOVA	Overall effect	57.61 v(4,10)	<0.0001
	Bonferroni post-hoc	CTRL vs LRRK2-PD-1	-	<0.0001
	Bonferroni post-hoc	CTRL vs LRRK2-PD-2	-	<0.0001
4J	One-way ANOVA	Overall effect	6.958 (4,10)	0.006
	Bonferroni post-hoc	CTRL vs LRRK2-PD-1	-	0.024
	Bonferroni post-hoc	CTRL vs LRRK2-PD-2	-	0.0362
4K	One-way ANOVA	Overall effect	14.02 (4,10)	0.0004
	Bonferroni post-hoc	CTRL vs LRRK2-PD-1	-	0.0054
	Bonferroni post-hoc	CTRL vs LRRK2-PD-2	-	0.0047

Table S7: Statistical summary of data in Figure 4C-K. Statistical significance was determined by one-way ANOVA followed by the Bonferroni's post-hoc test. Values indicate F values (F), degrees of freedom (df) and p values. $p < 0.05$ was considered statistically significant.

Figure	Analysis	Comparison	F(df)	Adjusted p-value
5C	One-way ANOVA	Overall effect	59.44 (4,10)	<0.0001
	Bonferroni post-hoc	CTRL vs LRRK2-PD-1	-	<0.0001
	Bonferroni post-hoc	CTRL vs LRRK2-PD-2	-	<0.0001

Table S8: Statistical summary of data in Figure 5C. Statistical significance was determined by one-way ANOVA followed by the Bonferroni's post-hoc test. Values indicate F values (F), degrees of freedom (df) and p values. $p < 0.05$ was considered statistically significant.

Figure	Analysis	Comparison	F(df)	Adjusted p-value
6B	One-way ANOVA	Overall effect	16.65 (4,10)	0.0002
	Bonferroni post-hoc	mDA+CTRL vs mDA+ LRRK2-PD-1	-	0.009
	Bonferroni post-hoc	mDA+CTRL vs mDA+ LRRK2-PD-2	-	0.0394
6C	One-way ANOVA	Overall effect	25.34 (4,10)	<0.0001
	Bonferroni post-hoc	mDA+CTRL vs mDA+ LRRK2-PD-1	-	0.0003
	Bonferroni post-hoc	mDA+CTRL vs mDA+ LRRK2-PD-2	-	0.0002
6E	One-way ANOVA	Overall effect	10.02 (4,10)	0.0016
	Bonferroni post-hoc	mDA+CTRL vs mDA+ LRRK2-PD-1	-	0.0226
	Bonferroni post-hoc	mDA+CTRL vs mDA+ LRRK2-PD-2	-	0.0215
6F	One-way ANOVA	Overall effect	21.58 (4,10)	<0.0001
	Bonferroni post-hoc	mDA+CTRL CM vs mDA+ LRRK2-PD-1 CM	-	0.0009
	Bonferroni post-hoc	mDA+CTRL CM vs mDA+ LRRK2-PD-2 CM	-	0.0017

Table S9: Statistical summary of data in Figure 6B-C and 6E-F. Statistical significance was determined by one-way ANOVA followed by the Bonferroni's post-hoc test. Values indicate F values (F), degrees of freedom (df) and p values. $p < 0.05$ was considered statistically significant.

Figure	Analysis	Comparison	F(df)	Adjusted p-value
7B	One-way ANOVA	Overall effect	29.53 (3,8)	0.0001
	Bonferroni post-hoc	mDA+LRRK2-PD-1 vs mDA+LRRK2-PD-1+ GSK	-	0.0005
	Bonferroni post-hoc	mDA+LRRK2-PD-2 vs mDA+LRRK2-PD-2+ GSK	-	0.0008
7C	One-way ANOVA	Overall effect	10.60 (3,8)	0.0037
	Bonferroni post-hoc	mDA+LRRK2-PD-1 vs mDA+LRRK2-PD-1+ GSK	-	0.0361
	Bonferroni post-hoc	mDA+LRRK2-PD-2 vs mDA+LRRK2-PD-2+ GSK	-	0.0192
7D	One-way ANOVA	Overall effect	6.490 (3,8)	0.0155
	Bonferroni post-hoc	LRRK2-PD-1 vs LRRK2-PD-1+ GSK	-	0.0196
	Bonferroni post-hoc	LRRK2-PD-2 vs LRRK2-PD-2+ GSK	-	0.0211
7E	One-way ANOVA	Overall effect	9.7 (3,8)	0.0048
	Bonferroni post-hoc	LRRK2-PD-1 vs LRRK2-PD-1+ GSK	-	0.0237
	Bonferroni post-hoc	LRRK2-PD-2 vs LRRK2-PD-2+ GSK	-	0.0465
7F	One-way ANOVA	Overall effect	27.03 (3,8)	<0.0001
	Bonferroni post-hoc	LRRK2-PD-1 vs LRRK2-PD-1+ GSK	-	0.0004
	Bonferroni post-hoc	LRRK2-PD-2 vs LRRK2-PD-2+ GSK	-	<0.0001
7G	One-way ANOVA	Overall effect	19.67 (3,8)	0.0005
	Bonferroni post-hoc	LRRK2-PD-1 vs LRRK2-PD-1+ GSK	-	0.0467
	Bonferroni post-hoc	LRRK2-PD-2 vs LRRK2-PD-2+ GSK	-	0.0012
7H	One-way ANOVA	Overall effect	63.4 (4,10)	<0.0001
BDNF	Bonferroni post-hoc	LRRK2-PD-1 vs LRRK2-PD-1+ GSK	-	<0,0001
	Bonferroni post-hoc	LRRK2-PD-2 vs LRRK2-PD-2+ GSK	-	0.0006
7H	One-way ANOVA	Overall effect	589 (4,10)	<0.0001

GDNF	Bonferroni post-hoc	LRRK2-PD-1 vs LRRK2-PD-1+ GSK	-	<0.0001
	Bonferroni post-hoc	LRRK2-PD-2 vs LRRK2-PD-2+ GSK	-	<0.0001
7H NGF	One-way ANOVA	Overall effect	41.93 (4,10)	<0.0001
	Bonferroni post-hoc	LRRK2-PD-1 vs LRRK2-PD-1+ GSK	-	0.0009
	Bonferroni post-hoc	LRRK2-PD-2 vs LRRK2-PD-2+ GSK	-	0.0002
7I	One-way ANOVA	Overall effect	9.549 (3,8)	0.0051
	Bonferroni post-hoc	LRRK2-PD-1 vs LRRK2-PD-1+ GSK	-	0.0367
	Bonferroni post-hoc	LRRK2-PD-2 vs LRRK2-PD-2+ GSK	-	0.0337
7J	One-way ANOVA	Overall effect	15.75 (3,8)	0.0010
	Bonferroni post-hoc	LRRK2-PD-1 vs LRRK2-PD-1+ GSK	-	0.0203
	Bonferroni post-hoc	LRRK2-PD-2 vs LRRK2-PD-2+ GSK	-	0.0037

Table S10: Statistical summary of data in Figure 7B-J. Statistical significance was determined by one-way ANOVA followed by the Bonferroni's post-hoc test. Values indicate F values (F), degrees of freedom (df) and p values. $p < 0.05$ was considered statistically significant.

Figure	Analysis	Comparison	F(df)	Adjusted p-value
8B	One-way ANOVA	Overall effect	32.89 (3,8)	<0.0001
	Bonferroni post-hoc	mDA+LRRK2-PD-1 vs mDA+LRRK2-PD-1+ MLi-2	-	0.0003
	Bonferroni post-hoc	mDA+LRRK2-PD-2 vs mDA+LRRK2-PD-2+ MLi-2	-	0.0018
8C	One-way ANOVA	Overall effect	13.32 (3,8)	0.0018
	Bonferroni post-hoc	mDA+LRRK2-PD-1 vs mDA+LRRK2-PD-1+ MLi-2	-	0.0208
	Bonferroni post-hoc	mDA+LRRK2-PD-2 vs mDA+LRRK2-PD-2+ MLi-2	-	0.0086
8D	One-way ANOVA	Overall effect	13.91 (3,8)	0.0015
	Bonferroni post-hoc	LRRK2-PD-1 vs LRRK2-PD-1+ MLi-2	-	0.0068
	Bonferroni post-hoc	LRRK2-PD-2 vs LRRK2-PD-2+ MLi-2	-	0.0192
8E	One-way ANOVA	Overall effect	14.96 (3,8)	0.0012
	Bonferroni post-hoc	LRRK2-PD-1 vs LRRK2-PD-1+ MLi-2	-	0.0254
	Bonferroni post-hoc	LRRK2-PD-2 vs LRRK2-PD-2+ MLi-2	-	0.0043
8F	One-way ANOVA	Overall effect	14.47 (3,8)	0.0022
	Bonferroni post-hoc	LRRK2-PD-1 vs LRRK2-PD-1+ MLi-2	-	0.0086
	Bonferroni post-hoc	LRRK2-PD-2 vs LRRK2-PD-2+ MLi-2	-	0.028
8G	One-way ANOVA	Overall effect	8.789 (3,8)	0.0065
	Bonferroni post-hoc	LRRK2-PD-1 vs LRRK2-PD-1+ MLi-2	-	0.0453
	Bonferroni post-hoc	LRRK2-PD-2 vs LRRK2-PD-2+ MLi-2	-	0.0360
8H BDNF	One-way ANOVA	Overall effect	59 (4,10)	<0.0001
	Bonferroni post-hoc	LRRK2-PD-1 vs LRRK2-PD-1+ MLi-2	-	<0.0001
	Bonferroni post-hoc	LRRK2-PD-2 vs LRRK2-PD-2+ MLi-2	-	<0.0001
8H GDNF	One-way ANOVA	Overall effect	126 (4,10)	<0.0001
	Bonferroni post-hoc	LRRK2-PD-1 vs LRRK2-PD-1+ MLi-2	-	<0.0001
	Bonferroni post-hoc	LRRK2-PD-2 vs LRRK2-PD-2+ MLi-2	-	<0.0001
8H	One-way ANOVA	Overall effect	20.23 (4,10)	<0.0001

NGF	Bonferroni post-hoc	LRRK2-PD-1 vs LRRK2-PD-1+ MLi-2	-	0.0112
	Bonferroni post-hoc	LRRK2-PD-2 vs LRRK2-PD-2+ MLi-2	-	0.0008
8I	One-way ANOVA	Overall effect	28.91 (3,8)	0.0006
	Bonferroni post-hoc	LRRK2-PD-1 vs LRRK2-PD-1+ MLi-2	-	0.0015
	Bonferroni post-hoc	LRRK2-PD-2 vs LRRK2-PD-2+ MLi-2	-	0.0121
8J	One-way ANOVA	Overall effect	9.306 (3,8)	0.0055
	Bonferroni post-hoc	LRRK2-PD-1 vs LRRK2-PD-1+ MLi-2	-	0.0401
	Bonferroni post-hoc	LRRK2-PD-2 vs LRRK2-PD-2+ MLi-2	-	0.0156

Table S11: Statistical summary of data in Figure 8B-J. Statistical significance was determined by one-way ANOVA followed by the Bonferroni's post-hoc test. Values indicate F values (F), degrees of freedom (df) and p values. $p < 0.05$ was considered statistically significant.

Figure	Analysis	Comparison	F(df)	Adjusted p-value
9B	One-way ANOVA	Overall effect	17.52 (5,12)	<0.0001
	Bonferroni post-hoc	LRRK2-PD-1 vs LRRK2-PD-1 + GSK	-	0.0002
	Bonferroni post-hoc	LRRK2-PD-1 vs LRRK2-PD-1 + MLi-2	-	0.0006
	Bonferroni post-hoc	LRRK2-PD-2 vs LRRK2-PD-2 + GSK	-	0.0134
	Bonferroni post-hoc	LRRK2-PD-2 vs LRRK2-PD-2 + MLi-2	-	0.0123

Table S12: Statistical summary of data in Figure 9B. Statistical significance was determined by one-way ANOVA followed by the Bonferroni's post-hoc test. Values indicate F values (F), degrees of freedom (df) and p values. $p < 0.05$ was considered statistically significant.

Figure	Analysis	Comparison	F(df)	Adjusted p-value
S1B	One-way ANOVA	Overall effect	11.15 (4,10)	0.0010
	Bonferroni post-hoc	CTRL vs LRRK2-PD-1	-	0.0161
	Bonferroni post-hoc	CTRL vs LRRK2-PD-2	-	0.0123
S1C	One-way ANOVA	Overall effect	133.5 (4,10)	<0.0001
	Bonferroni post-hoc	CTRL vs LRRK2-PD-1	-	<0.0001
	Bonferroni post-hoc	CTRL vs LRRK2-PD-2	-	0.0040

Table S13: Statistical summary of data in Figure S1B-C. Statistical significance was determined by one-way ANOVA followed by the Bonferroni's post-hoc test. Values indicate F values (F), degrees of freedom (df) and p values. $p < 0.05$ was considered statistically significant.

Figure	Analysis	Comparison	F(df)	Adjusted p-value
S3B	One-way ANOVA	Overall effect	30.91 (5,12)	<0.0001
	Bonferroni post-hoc	LRRK2-PD-1 vs LRRK2-PD-1 + GSK	-	0.0002
	Bonferroni post-hoc	LRRK2-PD-1 vs LRRK2-PD-1 + MLi-2	-	0.0003
	Bonferroni post-hoc	LRRK2-PD-2 vs LRRK2-PD-2 + GSK	-	0.0002
	Bonferroni post-hoc	LRRK2-PD-2 vs LRRK2-PD-2 + MLi-2	-	<0.0001
S3C	One-way ANOVA	Overall effect	310.4 (5,12)	<0.0001
	Bonferroni post-hoc	LRRK2-PD-1 vs LRRK2-PD-1 + GSK	-	<0.0001
	Bonferroni post-hoc	LRRK2-PD-1 vs LRRK2-PD-1 + MLi-2	-	<0.0001
	Bonferroni post-hoc	LRRK2-PD-2 vs LRRK2-PD-2 + GSK	-	<0.0001
	Bonferroni post-hoc	LRRK2-PD-2 vs LRRK2-PD-2 + MLi-2	-	<0.0001

Table S14: Statistical summary of data in Figure S3B-C. Statistical significance was determined by one-way ANOVA followed by the Bonferroni's post-hoc test. Values indicate F values (F), degrees of freedom (df) and p values. $p < 0.05$ was considered statistically significant.

Full Blot images

pSer935 LRRK2

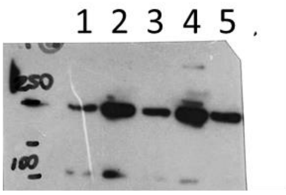

pSer106 Rab12

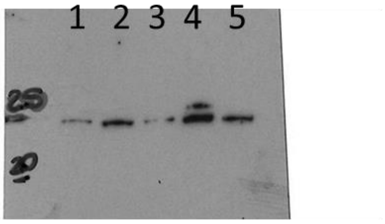

1. CTRL
2. LRRK2-PD-1
3. LRRK2-ISO-1
4. LRRK2-PD-2
5. LRRK2-ISO-2

Total LRRK2

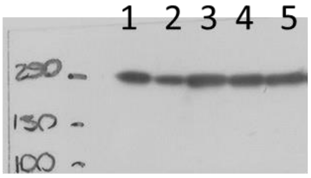

Total Rab12

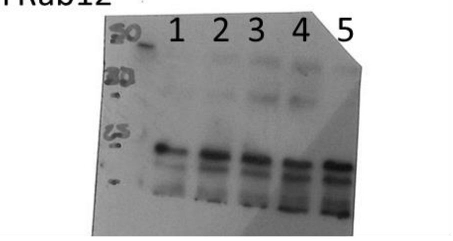

GAPDH

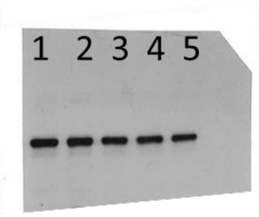

GFAP

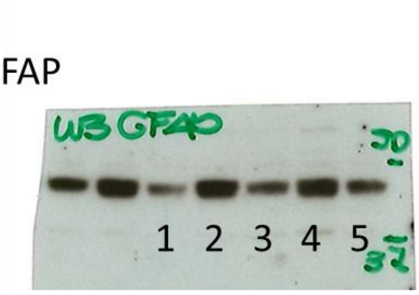

1. CTRL
2. LRRK2-PD-1
3. LRRK2-ISO-1
4. LRRK2-PD-2
5. LRRK2-ISO-2

GAPDH

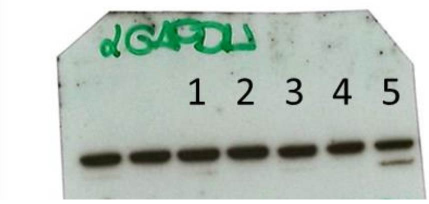

pSer935 LRRK2

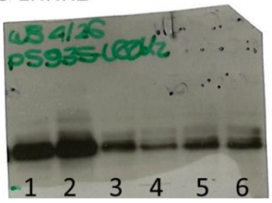

pSer106 Rab12

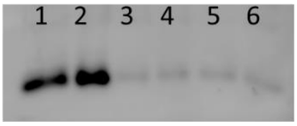

Total LRRK2

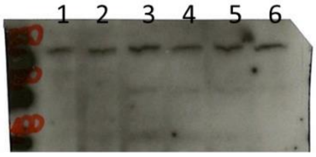

Total Rab12

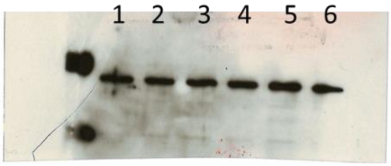

GAPDH

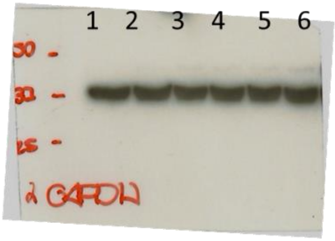

1. LRRK2-PD-1
2. LRRK2-PD-2
3. LRRK2-PD-1 + GSK
4. LRRK2-PD-2 + GSK
5. LRRK2-PD-1 + MLi-2
6. LRRK2-PD-2 + MLi-2

D2R

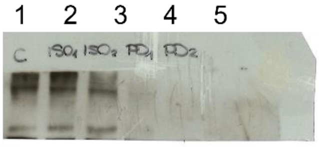

Na+K+-ATPase

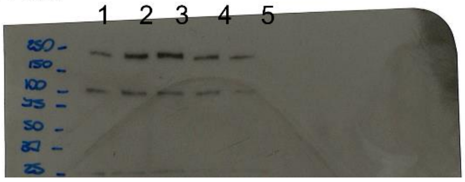

1. CTRL
2. LRRK2-ISO-1
3. LRRK2-ISO-2
4. LRRK2-PD-1
5. LRRK2-PD-2
